# *Stenotrophomonas maltophilia* Virulence and Specific Variations in Trace Elements during Acute Lung Infection: Implications in Cystic Fibrosis

**DOI:** 10.1371/journal.pone.0088769

**Published:** 2014-02-28

**Authors:** Arianna Pompilio, Domenico Ciavardelli, Valentina Crocetta, Ada Consalvo, Roberta Zappacosta, Carmine Di Ilio, Giovanni Di Bonaventura

**Affiliations:** 1 Clinical Microbiology Unit, Center of Excellence on Aging, “G. d'Annunzio” University Foundation, Chieti, Italy; 2 Department of Experimental and Clinical Sciences, School of Medicine, “G. d'Annunzio” University, Chieti, Italy; 3 School of Engineering, Architecture and Motor Science, “Kore” University, Enna, Italy; The Scripps Research Institute and Sorrento Therapeutics, Inc., United States of America

## Abstract

Metal ions are necessary for the proper functioning of the immune system, and, therefore, they might have a significant influence on the interaction between bacteria and host. Ionic dyshomeostasis has been recently observed also in cystic fibrosis (CF) patients, whose respiratory tract is frequently colonized by *Stenotrophomonas maltophilia*. For the first time, here we used an inductively mass spectrometry method to perform a spatial and temporal analysis of the pattern of changes in a broad range of major trace elements in response to pulmonary infection by *S. maltophilia*. To this, DBA/2 mouse lungs were comparatively infected by a CF strain and by an environmental one. Our results showed that pulmonary ionomic profile was significantly affected during infection. Infected mice showed increased lung levels of Mg, P, S, K, Zn, Se, and Rb. To the contrary, Mn, Fe, Co, and Cu levels resulted significantly decreased. Changes of element concentrations were correlated with pulmonary bacterial load and markers of inflammation, and occurred mostly on day 3 post-exposure, when severity of infection culminated. Interestingly, CF strain – significantly more virulent than the environmental one in our murine model - provoked a more significant impact in perturbing pulmonary metal homeostasis. Particularly, exposure to CF strain exclusively increased P and K levels, while decreased Fe and Mn ones. Overall, our data clearly indicate that *S. maltophilia* modulates pulmonary metal balance in a concerted and virulence-dependent manner highlighting the potential role of the element dyshomeostasis during the progression of *S. maltophilia* infection, probably exacerbating the harmful effects of the loss of CF transmembrane conductance regulator function. Further investigations are required to understand the biological significance of these alterations and to confirm they are specifically caused by *S. maltophilia*.

## Introduction

*Stenotrophomonas maltophilia* is a Gram-negative opportunistic pathogen especially in hospitalized or compromised patients where infection is associated to high mortality and morbidity [1], [2]. *S. maltophilia* has recently emerged as one of the most frequently found bacteria in cystic fibrosis (CF) patients [3], [4].

Despite increased *S. maltophilia* isolation from CF patients, its potential for pathogenicity remains undetermined because of conflicting clinical results from studies investigating the correlation between the presence of this microorganism and lung damage [5], [6]. However, in a series of studies we found evidences highly suggestive for the pathogenetic role of *S. maltophilia* in CF patients [7]–[13]. This microorganism is in fact able to grow as biofilms – sessile communities inherently resistant to antibiotics - not only on abiotic surfaces [7]–[10], but also on CF-derived epithelial monolayer [11], probably as a consequence of a selective adaptation to CF airways [12]. Furthermore, in a model of acute respiratory infection we observed that *S. maltophilia* significantly contributes to the inflammatory process resulting in compromised respiratory function and death [13].

It is well known that during an infectious process, along with immune system activation, a sequential series of alterations in metabolism occurs, including changes in trace element balance [14]–[16]. In fact, the host's acute response to infection generally includes increased synthesis of metal-binding proteins and a concomitant flux of trace elements between blood and tissues [16]. Metal ions are necessary for the proper functioning of the immune system, and, therefore, they might have a significant influence on the interaction between bacteria and host.

Ionic dyshomeostasis has also recently been observed in CF patients. Sputum and lavage fluid from CF patients infected with *P. aeruginosa* contain high concentrations of calprotectin [17], whose antimicrobial properties are mediated by the chelation of zinc [18], and increased total iron and iron-binding proteins levels [19], [20]. Gray et al. [19] suggested a relationship between high concentrations of total zinc and iron and airways inflammation both in CF and non-CF bronchiectasis, with longitudinal changes being observed in CF patients.

Although changes in some essential and non-essential trace elements have been previously described for some bacterial and parasitic pathogens [15], [21], [22], to the best of our knowledge no efforts have been made to evaluate the impact of *S. maltophilia* infection on trace element levels.

The main objective of the present study was, therefore, to evaluate the changes of a broad range of trace elements during the course of acute *S. maltophilia* lung infection. Moreover, we wanted to study whether these changes were associated to bacterial virulence.

To this, DBA/2 mouse lungs were comparatively infected by a strain collected from CF patient (Sm111), and one collected from an environmental source (C39). Mice were monitored during 7 days post-exposure (p.e.) for weight and health, pulmonary bacterial clearance, dissemination of infection, lung damage, and cytokine expression. Changes in trace elements were assessed, in both lungs and bronchoalveolar lavage (BAL), by Inductively Coupled Plasma Mass Spectrometry (ICP-MS) analysis, previously evaluated for its pre-analytical and analytical performances.

For the first time, here we demonstrated that *S. maltophilia* significantly affects the element profiles of both pulmonary tissue and BAL, and that these alterations strictly depend on bacterial virulence.

## Methods

### Ethics statement

This study was carried out in strict accordance with the recommendations in the Guide for the Care and Use of Laboratory Animals of the National Institute of Health. The protocol was approved by the Animal Care Committee of “G. d'Annunzio” University of Chieti-Pescara (Permit Number: 1989/09). Efforts were made to minimize suffering. Particularly, all surgery was performed under anaesthesia and animals were humanely euthanized when they showed clinical signs suggestive for moribund condition [23].

### *S. maltophilia* strains and growth conditions

Two *S. maltophilia* strains were used in all experiments: Sm111 strain, repeatedly isolated during a 2-years period from the sputum of a CF patient; and C39 strain, isolated from wastewater. Bacteria were stored at −80°C until use.

To prepare infectious doses for mice exposure, each *S. maltophilia* strain was grown under agitation (130 rpm) in 200 ml of Trypticase soy broth (OxoidSpA; Garbagnate M.se, Milan, Italy) at 37°C for 16 h, then harvested by centrifugation (4000 rpm, 10 min, 4°C). Pellets were then washed twice and resuspended in cold 1% PBS (Sigma-Aldrich Srl, Milan, Italy). Cell density was adjusted to about 1.0×10^11^ CFU/ml, and 8 ml of this suspension were nebulized in challenge procedure.

### Mouse model of acute lung infection

#### i) Mice

Eight-week-old male (n = 100; mean weight ± SD: 22.6±1.3 g) specific-pathogen-free DBA/2N inbred mice (Charles River Laboratories Italia, Calco, Italy) were used in all experiments. Animals were randomly assigned to each group (n = 10 mice), housed, bred and maintained in polycarbonate HEPA-filtered cages with a 12-h light/dark cycle, with *ad libidum* access to sterile acidified water and irradiated diet.

#### ii) Exposure to *S. maltophilia*

To simulate an airborne lung infection, a “home-made” aerosol dispersal system housed in a biosafety cabinet was used, as previously described [13]. Briefly, mice were exposed to nebulized inoculum for 60 min, followed by 5 min for cloud decay, and finally exposed for 5 min to UV irradiation for decontamination. As a control, mice were treated in the same way but exposed to PBS only.

#### iii) Experimental lay-out

Ten mice were allocated for each group (infected, and control uninfected mice) at each time point (days 1, 3, and 7 p.e.). Following infection, both groups were monitored daily for weight and behaviour, considering them to be unwell when a >10% weigh loss or ill appearance (ruffled coats, huddled position, lack of retreat in handler's presence) were observed.

On days 1, 3, and 7 p.e., mice were intraperitoneally anesthetized by Avertin 10 mg/ml (Sigma-Aldrich), then sacrificed by exanguation. Mice were observed for macroscopic pulmonary damage, according to the “four-point scoring system” proposed by Johansen et al. [24], then subjected to BAL. Mice were then perfused *in situ* with 0.9% NaCl via the heart. At each time point considered, in each group 8 animals were randomly selected to be undergone to bacterial count, cytokines measurement, and ICP-MS analysis, while the remaining 2 mice were assigned to histological analysis. At each time point, spleen was also collected and homogenized in sterile saline for evaluating bacterial dissemination.

### Microbiological assays

#### i) Lungs homogenization

For each mouse, both lungs were pooled and then homogenized (24000 g/min, 15 sec) - on ice in 2 ml of sterile saline containing protease inhibitors (cOmplete, EDTA-free; Roche Diagnostic SpA, Milan, Italy) and phenylmethanesulfonyl fluoride (PMSF; Sigma-Aldrich) - using an homogenizer (Ultra Turrax T8; Ika-Werke GmbH & Co. KG, Germany).

#### ii) Evaluation of pulmonary bacterial load and infection dissemination

Bacterial load was assessed in both lung homogenate and BAL samples by plating, in triplicate, 10-fold serial dilutions of each sample on Mueller-Hinton agar (Oxoid). Following 24 h-incubation at 37°C, the number of CFU was counted, then normalized according to the wet weight of lungs, averaged, and compared between groups. The initial bacterial deposition in the lungs was also assessed in homogenized lungs collected 1 h p.e. Dissemination of infection was evaluated, by viable count, in spleen samples collected at each time point considered.

#### iii) Cytokine measurements

Lung homogenates were centrifuged (1500×g, 4°C, 10 min), then supernatants were collected for measurement of cytokine levels (IFN-γ, MIP-2 IL-6, and TNF-α) by an ELISA system (R&D Systems Inc; Minneapolis, USA), according to the manufacturer's recommendations. The detection limit for IFN-γ, IL-6, TNF-α, and MIP-2 was <2.0, equal to 1.6, <5.1, and <1.5 pg/ml, respectively.

#### iv) Histopathological analysis

Following perfusion, lungs were immediately fixed in 10% neutral buffered formalin at RT. Each lung was then sectioned along its longitudinal axis obtaining tissue 3 µm-thickness sections from dorsal lung surfaces, throughout the entire tissue block and at regular intervals of 50 µm. Sections were stained with hematoxylin-eosin. The degree of inflammation was scored by using of “five-point system” proposed by Dubin et al. [25]. Ten fields/lung were evaluated at low (10×), and high (63×) magnification. Histopathological evaluation was performed blindly respect to the origin of the samples.

### ICP-MS analysis

#### i) Chemicals and reagents

Nitric acid (69%) and S stock (Na_2_SO_4_, 1000 mg/l, H_2_O) solutions were purchased from Romil (Cambridge, UK), while P stock solution (H_3_PO_4_, 1000 mg/l, H_2_O) was from Merck (Darmstadt, Germany). The multi-element calibration standard stock solution (Mg, Ca, Mn, Fe, Co, Cu, Zn, Se, Rb, and Sr at 10 mg/l; 2% HNO_3_), the tuning solution (Li, Y, Ce, and Tl at 10 mg/l; 2% HNO_3_), and the internal standard stock solution (Sc, Ge, and Y at 10 mg/l; 1% HNO_3_) were purchased from Agilent Technologies (Tokyo, Japan). Stock solutions and samples were diluted by deionised H_2_O with a specific resistance of 18.2 MΩ cm (Milli-Q system, Millipore, Watford, Hertfordshire, UK).

#### ii) ICP-MS operating conditions

ICP-MS analysis was performed by a 7500A ICP mass spectrometer (Agilent Technologies) fitted with an ASX-510 auto-sampler (Cetac Technologies, Omaha, NE), a peristaltic pump, a Babington nebulizer, and a Scott spray chamber (Agilent Technologies) as previously described [26], [27]. A detailed description of ICP-MS operating conditions can be found in Data S1.

#### iii) Samples

Lungs and BAL samples collected on days 1, 3, and 7 p.e. from both perfused sham-exposed (controls) and *S. maltophilia*-exposed mice were analysed by ICP-MS. Briefly, lung samples were homogenized, freeze-dried by using a Modulyo freeze-dryer (ThermoSavant, USA), accurately weighted by an AX26 DeltaRange™ balance (Mettler-Toledo, Greifensee, Switzerland), and then quantitatively transferred in sterile 15 ml-polystyrene tubes (BD Falcon, BD Biosciences, Franklin Lakes, NJ). Samples were dissolved in 0.8 ml of 65% (v/v) HNO_3_ (Romil) and 0.2 ml of 30% (v/v) H_2_O_2_ at 75°C for 5 h. The samples were diluted 1∶20 with 18 MΩ cm H_2_O, then analysed for Mg, P, S, K, Ca, V, Cr, Mn, Fe, Co, Cu, Zn, Se, and Rb. BAL samples were centrifuged and supernatants stored at −80°C until assayed. Samples were diluted 1∶10 in 18 MΩ cm deionised H_2_O and analysed for the same elements with exception of P, S, and K. Quantification was performed using the standard addition method and internal standard correction. Three spike levels were made on the basis of the specific element concentration. Samples showing evident blood contaminations were excluded. Limit of detection (LOD), limit of quantitation (LOQ), calibration functions, repeatability, trueness, and recovery function of ICP-MS analysis were assessed as reported in Data S1.

### Statistical analysis

All assays were carried out in triplicate and repeated on two different occasions. Differences among groups were evaluated using two-tailed Student's *t* test (to compare the element concentrations measured in both perfused and not perfused lung tissues) or one-way analysis of variance (ANOVA) followed by Tukey's multiple comparison *post-hoc* for parametric data (to evaluate the main effects on trace element levels of p.e. time and group, as well as the interaction between these factors), Kruskal-Wallis test followed by Dunn's multiple comparison *post-hoc* for nonparametric data, and chi-square test for percentages.

3×3 factor ANOVA followed by Tukey's multiple comparison *post-hoc* test was performed in order to evaluate the main effects of post-exposure time (1, 3, 7 days p.e.) and exposure to PBS, *S. maltophilia* SM111 or C39 strains, as well as the interaction between these factors and element concentrations in lung and BAL.

The assumption of homoscedasticity was verified by Levene test. Alignment and ranking were performed for heteroscedastic data before two-way ANOVA by using the ART web software available at http://faculty.washington.edu/aimgroup/proj/art/artweb [28].

Principal component analysis (PCA) was performed following the autoscaling of data that were normalized at mean  = 0 and SD = 1. Significance of correlations between elements and markers of infection was evaluated by Spearman correlation analysis of the combined data derived from the infected and control mice using MetaboAnalyst statistical analysis module [29]. Multiple testing corrections were performed by calculating the false discovery rate (FDR). Statistical analysis was performed by GraphPad 6.0 (GraphPad Software Inc., San Diego, CA, USA), Statistica 6.0 (Statsoft, Tulsa, OK), and XLStat (Microsoft, USA) software. Statistical significance was set at 95% confidence level.

## Results

### Clinical mice response to *S. maltophilia* acute lung infection

#### i) Mice health and weight

**Figure 1:**
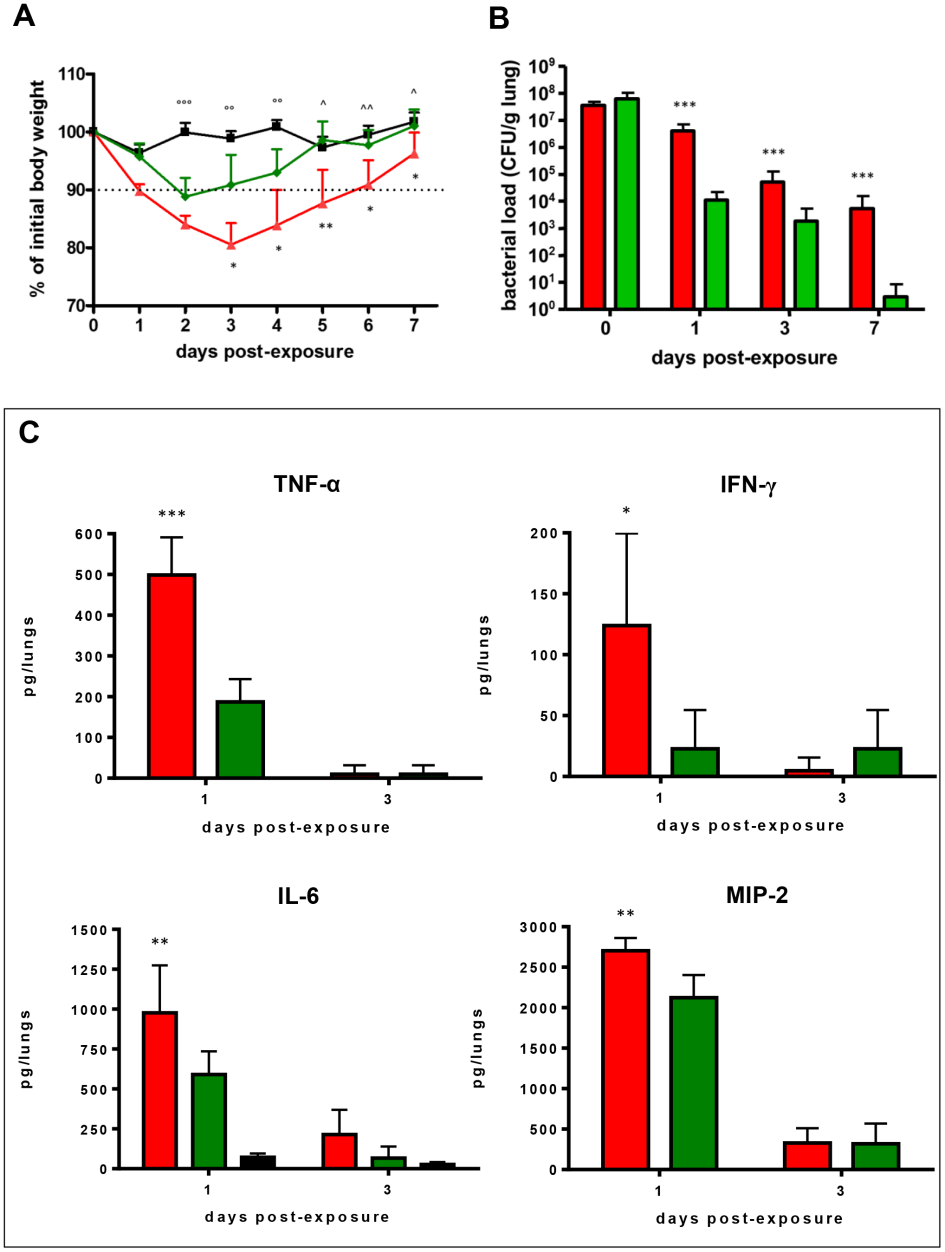
*S. maltophilia* acute lung infection in DBA/2 mice: weight, bacterial clearance, and cytokine levels. DBA/2 mice (n = 100) were exposed on day 0 to aerosolized *S. maltophilia* (CF Sm111 strain, red line or bars; environmental C39 strain, green line or bars) or PBS only (controls; black line or bars). A) Mice weight variations during the course of infection. The dotted line shows a 10% weight loss with regard to mean body weight before infection. Results were expressed as means + SDs. * p<0.05, ** p<0.01 for Sm111 vs C39; °°° p<0.001, °° p<0.01 for ctrl vs Sm111 and C39; ∧ p<0.05, ∧∧ p<0.01 for ctrl vs Sm111, chi-square test. B) Kinetic of DBA/2 mouse lung bacterial clearance. Following 1 h (day 0), and on days 1, 3, and 7 p.e. lungs were homogenized, then cultured for bacterial counts. Results were normalized to the lung wet weight (CFU/g) and shown as means + SDs. *** p<0.001 Sm111 vs C39, ANOVA followed by Tukey's multiple comparison *post-hoc* test. C) Cytokine levels measured in lung homogenates. Cytokines were assayed by ELISA on days 1, and 3 p.e. both in control and *S. maltophilia*-infected mice. Results were expressed as total content of cytokines in both lungs (pg/lungs) and shown as means + SDs. No detectable TNF-α, IFN-γ, and MIP-2 levels were found in lungs of control mice. * p<0.05, ** p<0.01, *** p<0.001, Sm111 vs C39, ANOVA followed by Tukey's multiple comparison *post-hoc* test.

The changes in body weight of infected and control mice over 7 days monitored, expressed as percentages of initial body weights, are shown in Figure 1A.

Compared to unexposed mice, the mean weight of mice infected by CF Sm111 strain showed to be significantly reduced from day 2 through day 7 p.e., whereas mice infected by environmental C39 strain significantly lost their body weight from day 2 through day 4 p.e. only (p<0.01).

With regard to strain type, Sm111-infected mice showed a mean body weight significantly lower than C39-infected ones from day 3 through day 7 p.e. (day 3: 80.6 vs 90.9%; day 4: 83.9 vs 93%; day 5: 87.7 vs 98.6%; day 6: 90.0 vs 97.7%; day 7: 96.3 vs 101%; p<0.05, except than for day 5, p<0.01). Mice infected with CF Sm111 strain lost more than 10% of their body weight from day 1 through day 6 p.e., and they did not completely regain weight during the period monitored. Contrarily, mice infected with environmental C39 strain showed a reduction higher than 10% on day 2 p.e. only, completely regaining weight on day 5 p.e. Control mice lost up to 5% of their body weight during the period monitored.

Contrary to control mice, infected ones usually showed symptoms suggestive for illness (slow responsiveness, and piloerection) from day 1 through day 7 p.e., regardless of strain tested.

#### ii) Infection clearance, dissemination, and mortality

Bacterial load was comparatively evaluated in lung tissue homogenates 1 h, and 1, 3, and 7 days p.e. Results, normalized to the wet lung weight and expressed as CFU/g, are summarized in Figure 1B.

The pulmonary deposition of *S. maltophilia* observed following 1 h p.e. was comparable for both strains (3.5±1.1 vs 6.3±3.9×10^7^ CFU/g, for Sm111 and C39 strains, respectively). Although bacterial load decreased from day 0 through day 7 p.e. regardless of strain tested, Sm111-infected mice retained significantly higher bacterial concentrations, regardless of time point considered (Sm111 vs C39: 4.1±2.8×10^6^ vs 1.9±1.1×10^4^ CFU/g, 5.0±7.7×10^4^ vs 1.8±3.5×10^3^ CFU/g, and 5.4±10.1×10^3^ vs 2.8±5.4 CFU/g, on days 1, 3, and 7 p.e., respectively; p<0.001) (Figure 1B). Control mice were negative for *S. maltophilia* at any time point.

To evaluate the invasiveness of *S. maltophilia*, we carried out microbiological analysis of spleen homogenates. The percentage of spleens positive for *S. maltophilia* was significantly higher in mice exposed to Sm111, compared to those infected with C39 (90 vs 10%, and 40 vs 0%, on days 1 and 3 p.e., respectively; p<0.001), although the bacterial counts were generally very low at each time point. On day 7 p.e., no spleens were positive for *S. maltophilia*, regardless of strain tested. Spleens from control mice were negative for *S. maltophilia*.

No mice were euthanized since signs suggestive for “moribund” status were not observed up to day 7 p.e. [23]. One *S. maltophilia* Sm111-infected mouse died on day 3 p.e. This animal couldn't be humanely euthanized because it died during the night. Mortality was not observed in control mice.

#### iii) Cytokine levels

Pulmonary TNF-α, IFN-γ, IL-6 and MIP-2 levels were assayed, in infected and control mice, on days 1 and 3 p.e., and results are reported in Figure 1C.

On day 1 p.e., all cytokines tested were found at levels significantly higher in Sm111-infected mice lungs than in C39-infected ones: TNF-α (498.8±92.4 vs 187.0±56.2 pg/lungs, respectively; p<0.001), IFN-γ (123.9±75.7 vs 22.7±31.8 pg/lungs, respectively; p<0.05); IL-6 (977.2±297.6 vs 590.0±145.8 pg/lungs, respectively; p<0.05), MIP-2 (2703.4±157.3 vs 2124.6±279.8 pg/lungs, respectively; p<0.01).

To the contrary, no difference in pulmonary levels of all cytokines assayed were found among groups on day 3 p.e. Therefore, pulmonary levels at day 7 p.e. were not assessed.

In control mice, none of the cytokines tested were detectable at the time points tested, except for IL-6 whose levels were significantly lower than those measured in infected mice, regardless of strain or time point considered (72.5±23.0 pg/lungs, and 26.9±15.0 pg/lungs on days 1 and 3 p.e., respectively; p<0.001).

#### iv) Macroscopic lung pathology

**Figure 2:**
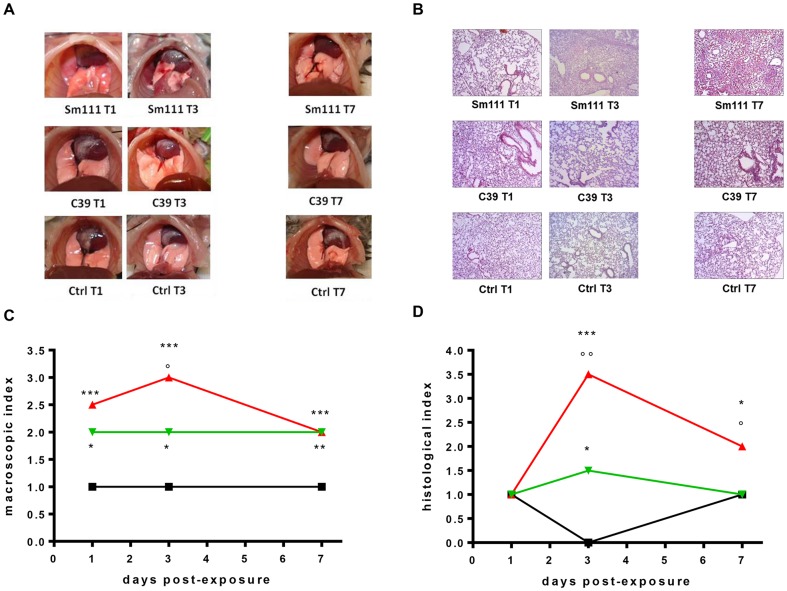
Assessment of lung damage in DBA/2 lung following infection with *S. maltophilia*. Lung damage was macroscopically and histologically assessed in DBA/2 mice (n = 18) on days 1, 3, and 7 p.e. to *S. maltophilia* (Sm111 CF strain, red line; C39 environmental strain, green line), or PBS only (control; black line). A–C) Macroscopic examination. A) Photographs of control and infected mouse lungs are representative for lung damage observed at each time point (days 1, 3, and 7 p.e.) in 8 mice per group. C) Lung damage was assessed by using “four-point scoring system” [24], and results are shown as median values. B–D) Histological assessment. B) Lung sections were stained with hematoxylin-eosin and ten fields/lung were observed at low (10×), and high (63×) magnification. For each group, only one representative microscopic field was reported. Since there were no significant differences in the degree of inflammation at any of the time point when evaluating the left and the right lung separately, the data were pooled and considered representative for both lungs. Mean percentage of tissue area characterized by the presence of neutrophilic infiltrate: 60% (Sm111-infected mice) vs 20% (C39-infected mice) (p<0.001, chi-square test.). D) Lung damage was quantified by using the “five-point scoring system” [25], and results are shown as median histopatological index. *** p<0.001, ** p<0.01, * p<0.05 vs ctrl; °° p<0.01, ° p<0.05 Sm111 vs C39; Kruskall-Wallis followed by Dunn's multiple comparison *post-hoc* test.

Macroscopic DBA/2 mouse lung damage, assessed by using the “four-point scoring system” [24], is summarized in Figures 2A and 2C.

At each time point monitored, *S. maltophilia*-infected lungs showed to be significantly more damaged compared to unexposed ones, regardless of strains considered (median scores, day 1 p.e.: 2.5 and 2 vs 1; day 3 p.e.: 3 and 2 vs 1; day 7 p.e.: 2 and 2 vs 1, for Sm111- and C39-exposed vs control mice, respectively; p<0.05).

Particularly, on days 1 and 7 p.e. infected lungs of both Sm111- and C39-exposed mice showed comparable damage (hyperemia and edema), although Sm111-exposed ones exhibited larger actelectasis, and consolidation. To the contrary, on day 3 p.e. Sm111-infected lungs were found to be significantly (p<0.05) more damaged than C39-infected ones, showing numerous abscesses, large atelectasis, and hemorrhage.

Control mice showed a score of 1 (normal lungs) throughout the study-period.

#### v) Lung histopathology

Histologic examination was carried out on lungs of both untreated and infected mice on days 1, 3 and 7 p.e. Representative sections illustrating lung inflammation for each group are shown in Figure 2B. In lungs of infected mice, we constantly observed a robust neutrophilic airway inflammation, irrespective of bacterial strain considered. However, Sm111-infected mice showed a more prominent area of inflammation than C39 mice did (60% vs 20%; p<0.001). Inflammation also involved in succession alveolar walls and sacs, and caused an increased septal thickening, oedema, and obliteration of most of alveolar spaces. Generally, lungs of untreated mice showed no evidence of inflammation. Necroscopy of Sm111-infected mouse died on day 3 p.e. revealed a marked neutrophilic inflammation involving about 90% of the alveolar compartment.

Microscopic DBA/2 mouse lung damage was also assessed by using a “five-point scoring system” [25], and results are summarized in Figure 2D. On day 1 p.e. no differences were observed among groups, all showing a median score of 1, suggestive for a normal lung. On day 3 p.e., although *S. maltophilia*-infected lungs showed a score significantly higher compared to unexposed control ones, damage caused by CF Sm111 strain resulted to be significantly higher than environmental C39 did (median score: 3.5 and 1.5 vs 0, for Sm111-, C39-exposed, and control mice, respectively; p<0.05).

This trend was also observed on day 7 p.e., when C39-exposed mice showed the same score of control mice (median score: 2.0, 1, and 1, for Sm111-, C39-exposed, and control mice, respectively; p<0.05).

### Changes in lung element profiles caused by *S. maltophilia* infection are dependent on strain and time

The analytical performances of ICP-MS element profiling of lung tissues fulfilled the requirement of quantitative analysis providing suitable sensitivity and accuracy for several analysed elements, although particular attention should be paid to remove blood contamination from lung tissue. A detailed description of analytical figures of merit and the effect of perfusion on pulmonary trace elements recovery by ICP-MS are shown in Data S1, Table S1, and Figure S1.

**Figure 3:**
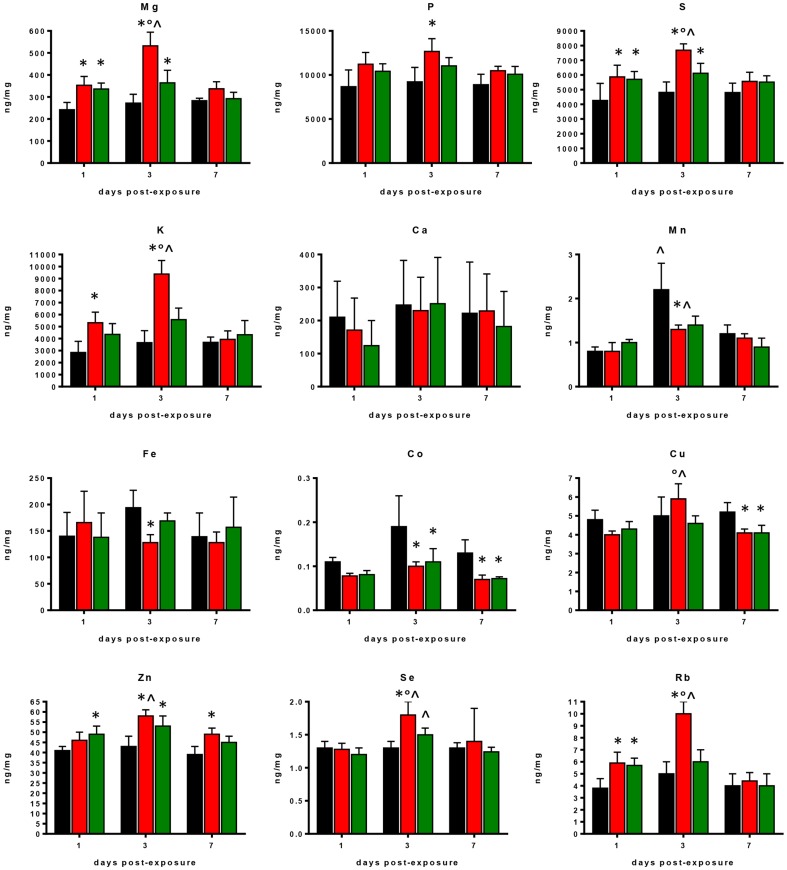
Changes in element profiles observed in lung tissues of control and *S. maltophilia*-infected DBA/2N mice. Amount of each element was assessed - on days 1, 3, and 7 p.e. to *S. maltophilia* (Sm111 CF strain, red bars; C39 environmental strain, green bars)or PBS only (controls, black bars) - by ICP-MS analysis in lung homogenates from DBA/2 mice (n = 10 each group). Results are expressed as ng of element for mg of lung tissue (dry weight), and graphed as means + SDs. ^*^ p<0.05, vs control mice; ° p<0.05, vs C39 strain; ^∧^ p<0.05, between days p.e.; two-way ANOVA followed by Tukey's post-hoc test.

Differences in major trace, and ultra-trace elements observed in lung tissue samples collected on days 1, 3, and 7 days p.e. to *S. maltophilia* or PBS (sham-exposed mice) are shown in Figure 3.

Two-factor ANOVA showed significant differences for all trace elements among groups considered, with the exception of Ca. Compared to sham-exposed mice, *S. maltophilia*-infected ones showed significantly higher pulmonary levels of Mg, P, S, K, Zn, Se, and Rb regardless of *S. maltophilia* strain. On the contrary, Mn, Fe, Co, and Cu concentrations were significantly reduced (Figure 3).

However, *S. maltophilia* strains tested exhibited markedly different behaviours. Particularly, Sm111 CF strain induced greater alterations compared to the environmental C39 strain, significantly increasing, on day 3 p.e., lung levels of Mg, S, K, Cu, Se, and Rb (mean ± SD, Mg: 532±62 vs 364±57 ng/mg; S: 7680±435 vs 6115±673; K: 9370±1133 vs 5582±958; Cu: 5.9±0.8 vs 4.6±0.4; Se: 1.8±0.2 vs 1.5±0.1; Rb: 10±1 vs 6±1, respectively; p<0.05) (Figure 3).

Furthermore, exposure to CF Sm111 strain significantly decreased Fe levels, while increased P levels on day 3 p.e., compared to controls (mean ± SD, Fe: 128±15 vs 194±33 ng/mg; P: 12674±1441 vs 9215±1635, respectively; p<0.05) (Figure 3). These effects were not observed in mice infected with C39 strain.

**Figure 4:**
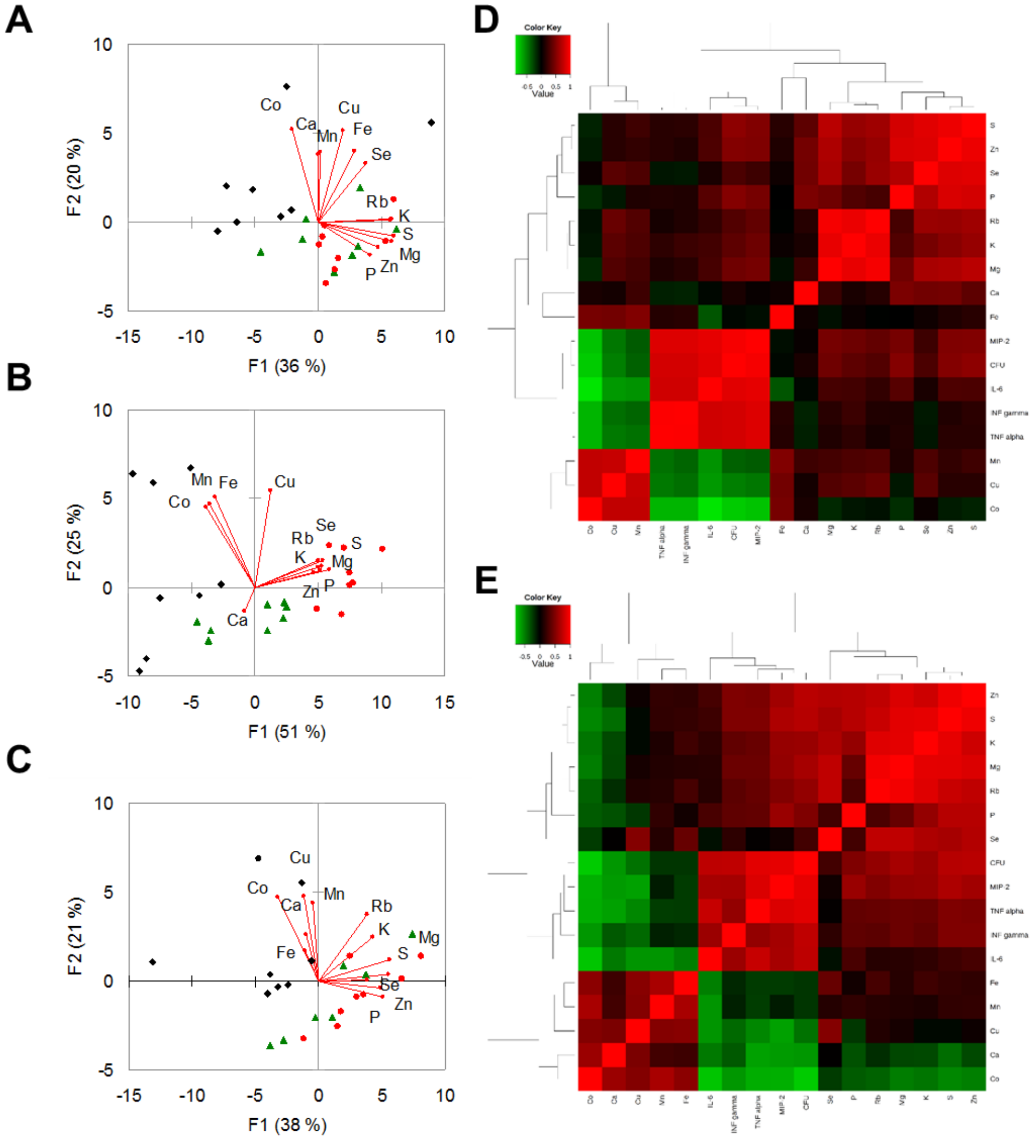
Trace elements, bacterial load, and cytokine levels observed in lung tissue of *S. maltophilia*-infected DBA/2N mice. A–C) Score and loading plots obtained by Principal Component Analysis. These plots show PC1 vs PC2summarizing 56%, 76%, and 59% of the variation among lung samples collected on days 1 (A), 3 (B), and 7 (C) p.e. to *S. maltophilia* (Sm111 CF strain, red circles; C39 environmental strain, green triangles) or PBS only (controls, black rhombi). The amounts of variance explained by each PC are shown in parentheses. D–E) Correlation maps. The heatmaps show the unsupervised hierarchical clustering of lung elements, bacterial counts in lung homogenate (CFU), and cytokines assessed using Spearman's correlation in response to *S. maltophilia* infection by Sm111 CF (D) or C39 (E) strain. Direct and inverse correlations are shown in red and green, respectively. Correlation and cluster analysis were performed using MetaboAnalyst statistical analysis module [29].

The unsupervised multivariate analysis by PCA shown in Figure 4 suggested that changes in element concentrations provoked by *S. maltophilia* infection were dependent on time p.e., and that these differences were higher on day 3 p.e. (Figure 4B).

Spearman correlation coefficient analysis showed significant correlations among analysed elements (Table 1), suggesting that changes of lung element concentrations in response to *S. maltophilia* infection are clustered in specific patterns. Particularly, Co levels were negatively correlated to Mg, S, and Zn levels (p<0.01).

**Table 1:** Correlations among trace elements, cytokines, and bacterial load observed in lung tissue from control and *S. maltophilia*-exposed DBA/2N mice.

Variable	Mg	P	S	K	Ca	Mn	Fe	Co	Cu	Zn	Se	Rb	IFN-γ	TNF- α	IL-6	MIP-2	CFU/lung
**Mg**	**1**																
**P**	**0,567** ***	**1**															
**S**	**0,786** ***	**0.791** ***	**1**														
**K**	**0,823** ***	**0.539** ***	**0.745** ***	**1**													
**Ca**	0,052	0.020	0.005	−0.041	**1**												
**Mn**	0,123	0.043	0.045	0.027	**0.309** ***	**1**											
**Fe**	−0.055	−0.091	0.089	0.011	0.028	**0.274** *	**1**										
**Co**	**−0.287** *	−0.205	**−0.282** *	−0.201	**0.250** *	**0.655** ***	**0.264** **	**1**									
**Cu**	**0.243** *	0.010	0.171	**0.331** **	0.211	**0.383** **	0.180	**0.474** ***	**1**								
**Zn**	**0.749** ***	**0.754** ***	**0.868** ***	**0.590** ***	0.117	0.118	0.049	**−0.292** *	0.158	**1**							
**Se**	**0.576** ***	**0.539** ***	**0.699** ***	**0.498** ***	0.205	0.155	0.231	−0.081	**0.448** ***	**0.685** ***	**1**						
**Rb**	**0.838** ***	**0.451** ***	**0.684** ***	**0.906** ***	0.033	0.091	−0.036	−0.123	**0.451** ***	**0.592** ***	**0.506** ***	**1**					
**IFN-γ**	0.176	0.110	0.178	0.199	−0.191	**−0.372** *	0.067	**−0.679** ***	**−0.395** *	0.180	−0.019	0.168	**1**				
**TNF-α**	0.111	0.101	0.155	0.142	−0.299	**−0.474** **	−0.007	**−0.710** ***	**−0.510** **	0.165	−0.133	0.123	**0.818** ***	**1**			
**IL-6**	0.230	0.246	0.247	0.208	−0.184	**−0.587** **	−0.286	**−0.802** ***	**−0.534** **	0.258	−0.010	0.212	**0.760** ***	**0.863** ***	**1**		
**MIP-2**	**0.365** *	**0.379** *	**0.418** *	0.359	−0.252	**−0.364** *	−0.103	**−0.752** ***	**−0.558** **	**0.418** *	0.049	0.322	**0.789** ***	**0.898** ***	**0.860** ***	**1**	
**CFU/lung**	**0.531** ***	**0.612** ***	**0.611** ***	**0.442** ***	−0.083	**−0.281** *	−0.116	**−0.455** ***	**−0.271** *	**0.576** ***	**0.271** *	**0.438** ***	**0.753** ***	***	**0.808** ***	**0.920** ***	**1**

Spearman coefficients calculated for significant correlations are shown in bold: ^*^ p<0.05, ^**^ p<0.01, ^***^ p<0.001. Correlations between pulmonary bacterial load (CFU/lung) and element concentrations were assessed by combining data sets from lung collected on days 1, 3, and 7 p.e. to PBS or *S. maltophilia* (Sm111 and C39 strains). Correlations between element concentrations and cytokines levels were calculated by combining data sets from lung collected on days 1 and 3 p.e. to PBS or *S. maltophilia* (Sm111 and C39 strains).

Strain-specific correlations among elements were also observed (Figure 4D; Table S2). Particularly, in Sm111-infected mice P was positively correlated to Mg, Se, and Rb levels, while Cu levels with Rb and K ones. A negative relationship instead was observed between P and Co, and between Mn and pulmonary bacterial load. In contrast, exposure to C39 strain resulted in a direct correlation between Ca and both Co and Cu levels.

### Changes in lung element concentrations correlate with the severity of infection

In order to tentatively investigate the biological significance of lung metal dyshomeostasis caused by *S. maltophilia* infection, we further evaluated the existence of a correlation between changes in lung element concentrations and markers for the severity of the infection, as assessed by pulmonary bacterial load (CFU/lung) and pro-inflammatory cytokine levels.

Overall, Spearman correlation analysis showed that several elements significantly correlated with pulmonary bacterial load (Table 1). Particularly, Mg, P, S, K, Zn, Se, and Rb levels positively correlated with bacterial load (p<0.001). To the contrary, Mn, Co, and Cu negatively correlated with CFU/lung (p<0.001 for Co; p<0.05 for Mn and Cu).

Statistically significant correlations were also found with cytokine levels (Table 1). Particularly, Mn, Co, and Cu levels were negatively correlated with all cytokines tested (p<0.001). To the contrary, Mg, P, S, and Zn levels were positively correlated to MIP-2 levels (p<0.001).

On the other hand, specific changes induced by exposure to C39 strain involved mainly cytokines. S and Zn levels were positively correlated to IFN-γ and TNF-α levels, while MIP-2 levels were directly correlated with K and Rb levels (Figure 4E; Table S3). To the contrary, a negative relationship was observed between Ca levels and both MIP-2, TNF-α, IL-6 levels, and pulmonary bacterial load.

### Changes in BAL element profiles caused by *S. maltophilia* infection are dependent on strain and time

To investigate the differences in ionomic profile between lung and BAL samples, the ICP-MS method developed for lung tissue was tentatively applied to measure trace element levels in BAL samples obtained before collecting lung samples. However, spike recovery experiments indicated the presence of a significant systematic error affecting ICP-MS analysis of BAL (data not shown). This is probably due to increased Na and Cl concentrations in BAL compared to lung samples, thus leading to higher matrix effect. The standard addition method with internal standard correction was, therefore, applied instead of external standard method. The sensitivity of the method was suitable for analysis of several elements with exception of Zn that was quantifiable only in few samples. In particular, Zn concentrations were not detectable in mice exposed to PBS and C39 strain. In contrast, Zn was measured in BAL collected on day 3 p.e. to Sm111 strain (21±4 µg/l).

**Figure 5:**
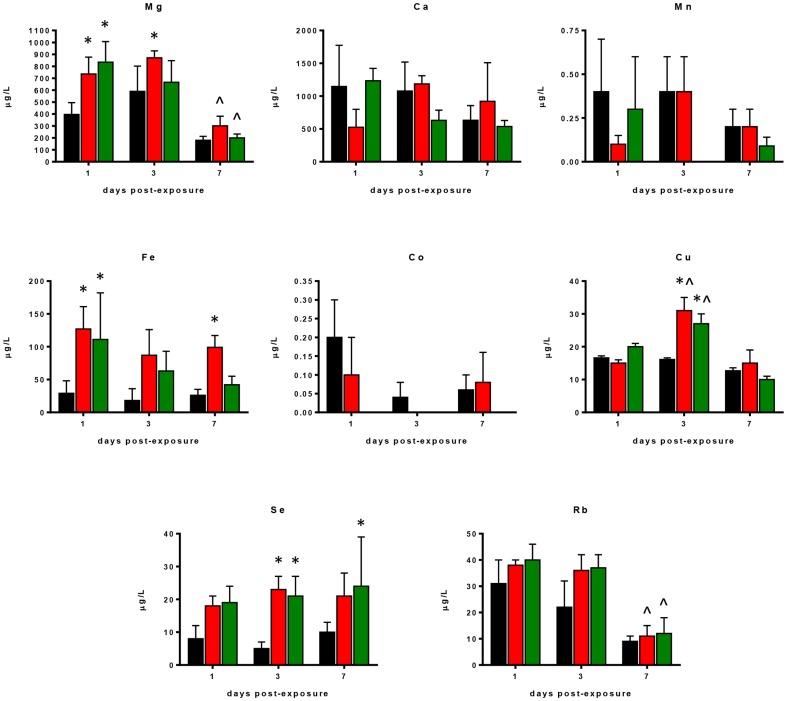
Changes in element profiles observed in BAL samples of control and *S. maltophilia*-infected DBA/2N mice. Amount of each element was assessed - on days 1, 3, and 7 p.e. to *S. maltophilia* (Sm111 CF strain, red bars; C39 environmental strain, green bars)or PBS only (controls, black bars) - by ICP-MS analysis in BAL from DBA/2 mice (n = 10 each group). Results are expressed as µg of element for liter of BAL, and graphed as means + SDs. ^*^ *p*<0.05, vs control mice. ^∧^ *p*<0.05, between days post-exposure; two-way ANOVA followed by Tukey's post-hoc test.

**Figure 6:**
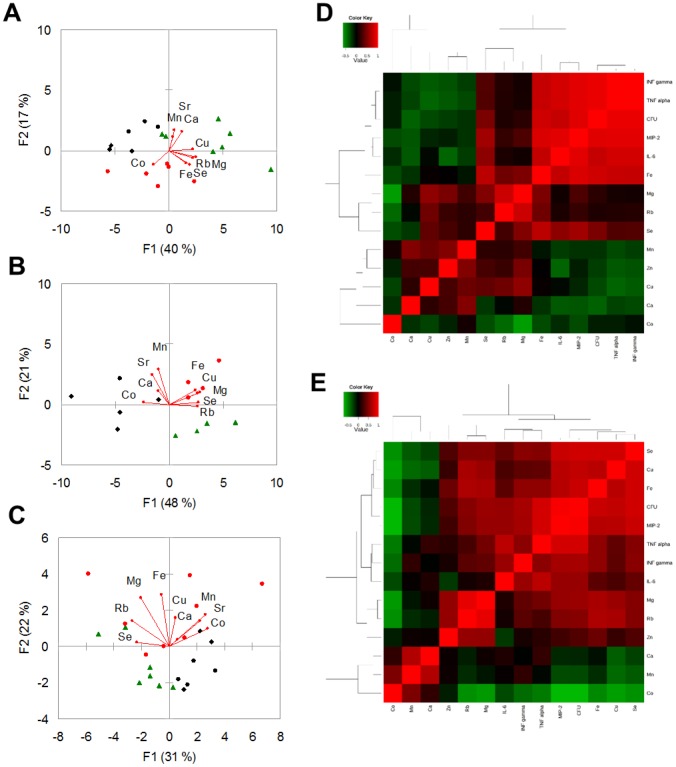
Trace elements, bacterial load, and cytokine levels observed in BAL samples of *S. maltophilia*-infected DBA/2N mice. A–C) Score and loading plots obtained by Principal Component Analysis. These plots show PC1 vs PC2 summarizing 57%, 69%, and 53% of the variation among BAL samples collected on days 1 (A), 3 (B), and 7 (C) p.e. to *S. maltophilia* (Sm111 CF strain, red circles; C39 environmental strain, green triangles) or PBS only (controls, black rhombi). The amounts of variance explained by each PC are shown in parentheses. D–E) Correlation maps. The heatmaps show the unsupervised hierarchical clustering of BAL elements, bacterial counts in lung homogenate (CFU), and pulmonary cytokines assessed using Spearman's correlation in response to *S. maltophilia* infection by Sm111 CF (D) or C39 (E) strain. Direct and inverse correlations are shown in red and green, respectively. Correlation and cluster analysis were performed using MetaboAnalyst statistical analysis module [29].

The differences in trace elements found in BAL samples collected on days 1, 3, and 7 p.e. to *S. maltophilia*, or PBS (sham exposed mice) are shown in Figures 5–6.

Two-way ANOVA showed significant differences in Mg, Fe, Cu, Se, and Rb levels among study groups. Particularly, *S. maltophilia*-infected mice showed increased pulmonary Mg, Fe, Cu, Se, and Rb levels compared to sham-exposed control mice (Figure 5).

Univariate data analysis and PCA indicated that changes in BAL ionomic profiles depended on strain and time (Figure 5; Figures 6A–B–C). In fact, Sm111 strain only caused higher Mg and Fe levels on days 3 and 7 p.e., respectively, compared to control mice (Mg: 871±58 vs 590±212 µg/l, Fe: 99±18 vs 26±9 µg/l, respectively) (Figure 5; Figure 6B). On the other hand, C39 strain increased Se levels on day 7 p.e. (24±15 vs 10±3 µg/l, respectively (Figure 5; Figure 6C). Furthermore, we found that Mg and Ca positively correlated with Cu and Zn, Fe positively correlated with Mn and Cu, while Co inversely correlated with Fe and Se regardless to the *S. maltophilia* strain (Table 2). These correlations were also found following the analysis for each *S. maltophilia* strain (Figures 6D–E; Table S4; Table S5), with minor differences.

**Table 2:** Correlations among trace elements found in BAL from control and *S. maltophilia*-exposed DBA/2N mice.

Variable	Mg	Ca	Mn	Fe	Co	Cu	Zn	Se	Rb	IFN-γ	TNF-α	IL-6	MIP-2	CFU/lung
**Mg**	**1**													
**Ca**	**0.393** **	**1**												
**Mn**	0.079	**0.535** ***	**1**											
**Fe**	**0.529** ***	0.114	0.057	**1**										
**Co**	**−0.304** *	−0.005	**0.385** **	−0.170	**1**									
**Cu**	**0.681** ***	**0.366** **	0.154	**0.386** **	−0.127	**1**								
**Zn**	**0.474** ***	**0.295** *	**0.294** *	0.115	−0.155	**0.500** ***	**1**							
**Se**	0.226	−0.200	**−0.358** **	**0.506** ***	**−0.487** ***	0.083	0.090	**1**						
**Rb**	**0.925** ***	0.221	−0.030	**0.435** **	−0.259	**0.561** ***	**0.385** **	0.174	**1**					
**IFN-γ**	0.103	−0.201	0.103	**0.698** ***	−0.058	−0.232	−0.278	0.237	0.098	**1**				
**TNF-α**	0.117	−0.095	−0.061	**0.701** ***	−0.118	−0.270	−0.288	0.223	0.081	**0.832** ***	**1**			
**IL-6**	0.100	−0.072	0.063	**0.633** ***	−0.126	−0.107	−0.316	0.331	0.024	**0.790** ***	**0.859** ***	**1**		
**MIP-2**	0.287	−0.215	−0.145	**0.770** ***	−0.347	0.024	−0.114	**0.507** **	0.197	**0.806** ***	**0.897** ***	**0.847** ***	**1**	
**CFU/lung**	**0.553** ***	−0.031	−0.028	**0.785** ***	−0.186	**0.399** **	0.122	**0.410** **	**0.444** **	**0.776** ***	**0.819** ***	**0.804** ***	**0.931** ***	**1**

Spearman rank correlation coefficients were calculated and significant correlations are shown in bold: ^*^ p<0.05, ^**^ p<0.01, ^***^ p<0.001. Correlations between pulmonary bacterial load (CFU/lung) and element concentrations were assessed by combining data sets from lung collected on days 1, 3, and 7 p.e. to PBS or *S. maltophilia* (Sm111 and C39 strains). Correlations between element concentrations and cytokines levels were calculated by combining data sets from BAL collected on days 1 and 3 p.e. to PBS or *S. maltophilia* (Sm111 and C39 strains).

### Changes in BAL element concentrations correlate with the severity of infection

As well as for lung samples, we evaluated relationship between the changes in BAL element levels and markers for the severity of the infection.

Regardless of strain considered, Spearman correlation analysis showed that several elements significantly correlated with pulmonary bacterial load or cytokine levels (Table 2). In particular, we found that Mg, Cu, and Rb positively correlated with bacterial load (CFU/lung). We also found a direct correlation between Se and both bacterial load and MIP-2, and between Fe and all markers of infection.

Furthermore, *S. maltophilia* strains significantly differed for correlation between elements and bacterial load or citokines (Tables S3–S4). Particularly, in Sm111-infected mice, Fe levels were positively correlated to all considered markers of infection, while Se levels correlated with bacterial load (p<0.001), IL-6 (p<0.05), and MIP-2 (p<0.001) concentrations (Figure 6D; Table S4).

With regard to C39 strain, bacterial load was positively correlated to Mg (p<0.01), Fe (p<0.01), Cu (p<0.01), Se (p<0.001), and Rb (p<0.01), while a negative relationship instead was observed with Co concentrations (p<0.01) (Figure 6E; Table S5). Referring to cytokines, Fe and Se levels were positively correlated to IFN-γ, TNF-α, and MIP-2 levels. A positive correlation with MIP-2 levels only was found for Mg (p<0.01), Cu (p<0.01), and Rb (p<0.05) levels. To the contrary, a negative relationship was observed between Co levels and MIP-2, and TNF-α levels (p<0.01, and p<0.05; respectively).

### BAL element concentrations correlate with lung element levels

In order to assess whether BAL element alterations may correlate to lung metal dyshomeostasis, we assessed the correlation between the ionomic profiles found in lung and BAL samples.

We found a significant direct correlation between Mg levels measured in the lung and those in BAL. The same correlation was found for Mn (Table 3). To the contrary, Fe levels measured in lung and BAL samples were inversely correlated (Table 3).

**Table 3:** Correlations between elements in lung tissue and BAL from control and *S. maltophilia*-exposed DBA/2N mice.

	BAL							
	Element	Mg	Ca	Mn	Fe	Cu	Se	Rb
	**Mg**	**0.294** *°	−0.047	−0.052	**0.376** **	**0.390** **	**0.394** **	0.176
	**Ca**	−0.067	0.043	0.180	−0.045	0.167	−0.142	−0.123
	**Mn**	−0.081	0.033	**0.273** * °	**−0.277** *	0.084	−0.148	−0.154
**LUNG**	**Fe**	−0.005	−0.070	0.148	**−0.319** * °	−0.207	−0.138	−0.003
	**Cu**	−0.184	0.074	0.234	**−0.463** ***	0.091	**−0.415** **	−0.216
	**Se**	0.202	0.194	0.239	0.243	**0.372** **	0.228	0.052
	**Rb**	0.259	−0.014	0.009	0.179	**0.316** *	0.127	0.175

Spearman correlation coefficients were calculated and significant correlations are shown in bold: ^*^ p<0.05, ^**^ p<0.01, ^***^ p<0.001.

° Significant correlation between lung tissue and BAL levels for the same element.

With regard to strain type, exposure to CF Sm111 strain was associated to a positive correlation for Mg, Mn, and Se, while a negative correlation was observed for Fe (Spearman correlation coefficient: 0.346, 0.410, 0.404, and −0.357, respectively; p<0.05) (Table S6; Figure S2). On the contrary, exposure to environmental C39 strain caused a positive correlation for Co only (Spearman correlation coefficient: 0.568; p<0.001) (Table S7).

## Discussion

Although changes in plasma concentrations of several trace elements during various infections have been reported [14]–[16], [21], [22], the information on the concomitant changes in trace element balance in the target tissues of the infectious organism is limited. The assessment of local availability of trace elements in an infected tissue could be important since being part of systems regulating basic cell functions and essential to immune cell function, they might affect infection outcome.

In this study, for the first time we used ionomic approach to perform a spatial and temporal analysis of the pattern of changes in a broad range of both essential and non-essential trace elements in response to pulmonary infection by *S. maltophilia*. To this, ICP-MS was carried out, in both lung and BAL samples, to assess changes in ionomic profiles provoked, throughout 7 days, by *S. maltophilia* in a mouse model of acute lung infection we recently described [13].

The main finding of the present study was that pulmonary element profile is significantly affected by *S. maltophilia* infection, depending on post-exposure time and bacterial strain.

Increased lung levels of Mg, P, S, K, Zn, Se, and Rb were observed. In contrast, Mn, Fe, Co, and Cu levels were significantly decreased in lungs following exposure to *S. maltophilia*. The most consistent response to infection was, however, observed with Mg, S, and Co since their levels significantly changed regardless of strain tested and with greater persistence.

To tentatively correlate the observed changes in trace elements with the severity of lung infection, it is worthy of note that, as suggested by PCA analysis, most relevant changes in element concentrations caused by *S. maltophilia* infection occurred on day 3 p.e., that is when the severity of infection - in terms of body weight loss and lung damage - culminated in our murine model.

Furthermore, levels of all trace elements - except than for Fe, Ca, and Se - were significantly correlated with pulmonary bacterial load, thus suggesting that the observed changes are due to the host redistribution of elements in response to infection. On the other hand, it is improbable that these changes reflect bacterial ion content, considering that the contribution of bacterial biomass to samples tested is probably negligible.

Although the biological significances of these alterations are far to be completely understood and surely requires further investigations, some speculations can be made on the bases of our findings.

The increase in Zn, Mg, and Se levels is probably involved in the immunological host response, macrophage recruitment, and neutrophil infiltration, as suggested by the positive correlation we found with bacterial concentration and MIP-2 levels.

Zinc is a nutritional component necessary to the normal development and maintenance of immunologic functions related to T lymphocytes [29], and its levels in the host are increased during inflammation [30], [31]. There is increasing evidence suggesting that the host actively sequesters Zn during infection to hinder microbial growth. Consistent with our results, increased Zn in host tissues and fluids during *P. aeruginosa* chronic infection and inflammation has been reported [19], [32], [33]. Particularly, Gray et al. [19] recently found sputum Zn levels significantly higher in CF and non-CF bronchiectasis compared with chronic obstructive pulmonary disease and healthy control, while Griese et al. [33] observed higher Zn levels in exhaled air condensate of CF patients.

The increased Zn levels we observed may be consequence of a passive release by neutrophils, since they are rich in this element [34]. Gray et al. [19] observed higher sputum Zn levels correlated with calprotectin, MPO, and IL-8. Accordingly, we found that levels of Zn and MIP-2, a chemoattractant for neutrophils both *in vitro* and *in vivo* [35], were positively correlated. Furthermore, we observed most of lung tissue area was characterized by the presence of an abundant neutrophilic infiltrate, especially in the case of Sm111 strain-infected mice. A contribution to higher Zn levels could be also result from circulation during inflammation.

Similarly to Zn, Mg and Se are as well modulators of the immune system and increased levels in infected tissue have been reported [29], [36]. Increased Se concentrations could be related to the accumulation of immune cells and up-regulation of seleno-proteins, particularly those with antioxidant function [37]. To the contrary, higher Mg levels in infected tissues cannot be completely explained on the basis of immune cell infiltrate, as suggested by the positive correlation with MIP-2 levels we observed. In fact, several studies reported that immune and anti-inflammatory responses are stimulated by Mg deficiency, and decreased serum Mg levels have been related to immune cells proliferation [38].

Necrotizing inflammation we observed during *S. maltophilia* infection is very likely the cause for higher Rb levels, since this element is mainly deposited intracellularly.

In partial agreement with previous results [31], *S. maltophilia* infection also caused significant reduction of Co and Cu lung levels, furtherly corroborated by the negative correlation found between these elements and pulmonary bacterial load. Interestingly, all markers of infection we considered were negatively correlated to Co and Cu levels, thus suggesting an efflux/recruitment of these elements from lung tissue in response to the bacterial infection.

In order to individuate the presence of ionomic profiles specifically associated to *S. maltophilia* virulence, changes in trace elements provoked in DBA/2N mice by Sm111 strain, isolated from a CF patient chronically infected, were comparatively evaluated to those observed following exposure to C39 strain, isolated from wastewater.

Our murine model of acute lung infection clearly showed that Sm111 CF strain was significantly more virulent than the environmental one, probably reflecting an adaptive behaviour of the microorganism during chronic infection that enables it to survive to the environmental stresses that are likely to be encountered within the habitat of the CF lung [12].

Particularly, Sm111 strain showed to be more efficient in persisting and invading murine airways, thus allowing dissemination of infection. Exposure to Sm111 also caused a lung damage significantly more severe than C39 did, as suggested by both macroscopic and histological examinations, and higher pro-inflammatory effects, as showed by cytokine pulmonary levels measurement.

It is interesting to highlight that the differential element profiles showed that *S. maltophilia* Sm111 CF strain significantly differed from C39 environmental one in pulmonary trace elements dyshomeostasis. Particularly, lung levels of Mg, S, K, Cu, Se, and Rb were significantly increased by Sm111 CF strain on day 3 p.e., compared to C39 strain.

It is worthy of note that exposure to Sm111 CF strain exclusively increased P and K lung levels, while decreased Fe and Mn ones. Manganese is another important trace metal required in numerous cellular processes, including metabolism and oxidative stress defense [39]. A recent work has demonstrated that in addition to Zn, vertebrates sequester Mn and Fe both intracellularly and extracellularly to protect themselves against infections [40]. Our results are consistent with these findings, although Sm111 CF strain probably provoked Fe and Mn dyshomeostasis by different mechanisms since only Mn levels negatively correlated with all markers of infection considered.

Correlation analysis among elements, and between elements and markers of infection also showed the existence of relationships shared by both strains, while others resulted to be exclusive for one strain only.

Taken together, our findings clearly suggest the existence of complex interactions among elements, and that *S. maltophilia* infection modulates lung metal balance in a concerted and strain-dependent manner. Essential trace elements are not only important for the host immune defense but also for microorganisms. Some elements - such as Zn, Cu, Mn, and Fe - are in fact essential components of many proteins and co-factors in enzymatic reactions [41], [42]. However, above certain concentrations, they can be also toxic to bacterial cells. Maintenance of intracellular homeostasis of metal ions is, therefore, crucial for survival of bacteria, and therefore they have developed several systems to monitor and control trace element levels, especially in certain environments as they can modulate the expression of genes necessary for colonization and virulence [43]–[47]. In this regard, some trends in trace elements we observed might significantly increase *S. maltophilia* virulence and, probably, its ability in establishing infection in CF lung. It has in fact reported that in *S. maltophilia* increased concentrations of Mg caused higher resistance levels to aminoglycosides, penicillins, quinolones, and cotrimoxazole [48]. Furthermore, RNase activity of *S. maltophilia* resulted to be inhibited by Co, while stimulated by Mg [49].

That said, the extensive flow of trace elements, in response to bacterial infection, is also aimed at affecting virulence potential of invading microorganisms. In that regard, the concerted changes in some trace elements we observed – namely, increased Zn levels, decreased Fe levels, and constant Ca levels – might represent a combined host defense against infection, since under these conditions haemolytic activity of *S. maltophilia* is significantly inhibited [50]. Further studies have to be performed using an adequate number of strains to fully evaluate the contribution of these changes to pathogenesis, and whether they were associated to virulence degree or, rather, related to the biological inter-strain variability.

During the course of most infections, an extensive adjustment in host metabolism occurs and trace elements are simultaneously redistributed, carried by acute-phase metal-binding proteins (i.e. Fe-binding ferritin, and the Cu-binding ceruloplasmin), among organs/compartments involved by the disease process [51]. Therefore, the ionomic profile of BAL samples was also assessed and comparatively evaluated with that observed in lung homogenates.

Spearman correlation coefficients analysis showed that, during *S. maltophilia* infection, lung and BAL levels were positively related in the case of Mg and Mn. On the contrary, increased Fe levels in BAL were associated to a simultaneous decrease of this element in the lung.

Iron is an essential micronutrient for both microbial pathogens and their mammalian hosts. Changes in Fe availability and distribution have significant effects on pathogen virulence and on the immune response to infection [52]. In agreement with our findings, increased Fe levels in sputum have been described in CF patients [20], possibly contributing to the proliferation of bacteria [53]. The inverse relationship we observed between lung and BAL Fe levels might, therefore, be an adaptive response intended to deprive invading pathogens of iron.

On the other hand, it is also possible to hypothesize that *S. maltophilia* might affect Fe efflux from tissues by competing with host for iron uptake, particularly ferrous iron. In this regard, the major route for bacterial ferrous iron uptake, namely Feo (Ferrous iron-transport) system, has been recently characterized also in *S. maltophilia* [54]. This hypothesis is further supported by the significant positive correlation we observed, in BAL samples, between Fe levels and bacterial load. In accordance to this trend, lung Fe levels also inversely correlated with bacterial load, even if did not reach the significance.

Stratifying for strain type, trends observed for Fe, Mg, and Mn were observed in CF Sm111 strain-infected mice only, also with addition of a positive correlation for Se. To the contrary, exposure to environmental C39 strain caused a positive correlation between compartments for Co levels only.

It is also interesting to note that Sm111 CF strain showed a more significant effect and a higher degree of correlation between Fe levels and bacterial load observed in BAL samples, thus suggesting its higher iron scavenging ability compared with the environmental isolate.

These findings support our previous hypothesis that - during *S. maltophilia* infection - Mg, Mn, and Fe lung dyshomeostasis are regulated by different and strain-dependent mechanisms. Particularly, our results were highly suggestive for an association between virulence and the specific recruitment of Mg, Mn, and Se, from lung parenchyma toward epithelial surface. Evaluation of these elements in BAL samples could be, therefore, helpful in assessing *S. maltophilia* virulence and, consequently, lung damage.

This may be of particular interest in CF research field. In fact, an adequate assessment of cytology and protein biomarkers (i.e. cytokines, chemokines) in sputum is critical for understanding and managing CF, as well as other inflammatory lung diseases. In this regard, several studies have focused on measurement of cytokines to assess levels of airways inflammation. Accordingly, we observed that *S. maltophilia* pulmonary concentration and cytokines (IL-6, MIP-2, IFN-γ, and TNF-α) expression were significantly correlated. The fluid phase of the induced sputum also contains nonorganic elements that might be considered as marker of lung pathophysiology, thus allowing more reliable measurement of inflammation in the airway compared to cytokines whose activity is significantly affected by protease activity in sputum [55]. However, our findings suggested that BAL ionomic profile did not exactly reflect lung physiological status, in terms of trace elements profile. Therefore, BAL samples might be partially suggestive for lung dyshomeostasis and, consequently, for monitoring the development of pulmonary tissue damage during infection.

Furthermore, in CF patients, trace elements balance is also influenced by impaired chloride efflux in CF airway epithelium, the most important hallmarks of CF [19], [20], [33], [56], [57]. In particular, specific mutations of CF transmembrane conductance regulator (CFTR) significantly affected metal homeostasis *in vitro* [58]. Furthermore, dyshomeostasis of K, Zn and NO_3_^−^ was observed in exhaled air condensate in CF patients [33], while increased levels of Cu, total Fe, and Fe-binding proteins were also found in CF sputum [19], [20].

For these reasons, restoring physiological lung element balance has been proposed as a promising new therapeutic approach to CF patient. Increasing the airway surface liquid Mg concentration by aerosolized or oral Mg supplementation might improve the removal of highly viscous mucus in chronic lung disease by activating endogenous DNase activity [59]. Abdulhamid et al. [60], recently observed that oral intake of Zn reduced the number of days of oral antibiotics used to treat respiratory infections in children with CF. Supplementation of Se in CF patients has been also proposed to maintain the oxidant-antioxidant balance, so improving lung function [61].

Furthermore, trace elements might affect CFTR functionality. In this regard, Li et al. [62] recently identified extracellular pseudohalide anions – namely Co(CN)_6_^3−^, Co(NO_2_)_6_^3−^, Fe(CN)_6_^3−^, IrCl_6_^3−^, and Fe(CN)_6_^4−^ - as increasing CFTR function.

Taken together, these observations, added to our results, further highlight the importance of a careful management of trace element balance in CF patients, especially during lung infections.

## Conclusions

Our results demonstrated that ICP-MS analysis represents an useful technique to evaluate changes in host ionomic profile during pulmonary infection, in order to understand the impact infection might have on the host. This approach might be particularly relevant to the management of lung infections in CF patients for the following reasons:

- it might inform future molecular studies on the interaction between microorganisms and the host regarding competition for specific mineral nutrients. Measurement in trace elements might in fact increase the understanding of the pathophysiological processes during pulmonary infection. This is particularly relevant for *S. maltophilia*, since bacterium-host interactions are not yet fully understood. Specifically, our findings showed that *S. maltophilia* uses the common strategy of modulating the host ionome, causing significant differences in trace elements balance, probably by direct or indirect mechanisms. The higher impact we observed on cationic homeostasis provoked by the more virulent Sm111 CF strain might be due to its adaption to a compromised environment, i.e. CF lung, and to be associated to an exacerbation of a particular step of pathogenesis.
- considering the potential association we observed between trace element levels and lung infection/inflammation, combined with their likely higher resistance to protease degradation compared to cytokines, trace elements could be considered as potentially robust markers of lung pathophysiology, thus offering new insight into pulmonary inflammation. Particularly, the observed changes in Mn, Mg, and Se levels in BAL samples seem to be exhaustively predictive for the severity of *S. maltophilia* lung infection. Therefore, their measurement in sputum might have potential diagnostic and prognostic usefulness.
- it might be valuable in defining/designing disease management strategies in CF patients. Control of CF disease includes managing nutritional status, as well as using of therapeutic modulators of CFTR function. Therefore, the observed adverse effect caused by *S. maltophilia* infection on trace elements status emphasizes the importance of careful monitoring and, if necessary, supplementation of oligoelements during therapy in CF patients concomitantly suffer from infections. This might otherwise jeopardise the outcome of treatment in the patient.

Further studies are warranted to confirm that changes in ionomic profile we observed are specifically caused by *S. maltophilia*, rather than they represent a consequence of infection-induced changes in host metabolism.

## Supporting Information

Data S1 Methods and Results.
(DOCX)

Figure S1 Effect of perfusion on pulmonary trace elements recovery by ICP-MS. Lungs of DBA/2 mice (n = 10) lungs were collected following perfusion with 0.9% NaCl (green bars). Control mice (n = 10) were not perfused (red bars). Results are means + SDs. * p<0.05, ** p<0.01 vs perfused lungs, two tailed Student's t-test.
(DOCX)

Figure S2 Correlation between Mg, Fe, and Se levels quantified in lung and BAL samples from *S. maltophilia*-infected DBA/2N mice. A, B, C) mice infected with CF Sm111 strain; D, E, F) mice infected with environmental C39 strain. Significance of correlations was assessed by Spearman correlation analysis. p values were: 0.039, 0.033, and 0.002 for Mg (A), Fe (B), and Se (C), respectively; 0.381, 0.492, and 0.850 for Mg (D), Fe (E), and Se (F), respectively (see also Tables S6 and S7).
(DOCX)

Table S1 ICP-MS analysis of mouse lung tissue: analytical figures of merit. The m/z measured for quantification, the internal standard (IS), the values of limit of detection (LOD) and limit of quantization (LOQ), the external calibration functions, precision and trueness are shown. Spike levels were: 20, 50, and 200 µg/l for Mg; 300, 500, and 1000 µg/l for P, S, and K; 20, 50, and 100 µg/l for Ca and Fe; 5, 20, and 100 µg/l for Cu and Rb; and 1, 5, and 10 µg/l for Mn, Co, and Se. ^a^ µg/l (µg/g); ^b^ µg/l; ^c^ CI  =  confidence interval, 95% confidence level; ^d^ 95% confidence level; ^e^ Coefficient of variation (%); ^f^ Standard deviation; ^g^ Relative standard deviation (%).
(DOCX)

Table S2 Correlations among elements, cytokines, and bacterial load observed in lung tissue of DBA/2N mice exposed to PBS or CF Sm111 *S. maltophilia* strain. Spearman rank correlation coefficients were calculated on data collected on days 1, 3, and 7 p.e. Significant correlations are shown in bold. * p<0.05, ** p<0.01, *** p<0.001.
(DOCX)

Table S3 Correlations among elements, cytokines, and bacterial load observed in lung tissue of DBA/2N mice exposed to PBS or environmental C39 *S. maltophilia* strain. Spearman rank correlation coefficients were calculated on data collected on days 1, 3, and 7 p.e. Significant correlations are shown in bold. * p<0.05, ** p<0.01, *** p<0.001.
(DOCX)

Table S4 Correlations among elements, cytokines, and bacterial load observed in BAL from DBA/2N mice exposed to PBS or CF Sm111 *S. maltophilia* strain. Spearman rank correlation coefficients were calculated on data collected on days 1, 3, and 7 p.e. Significant correlations are shown in bold. * p<0.05, ** p<0.01, *** p<0.001.
(DOCX)

Table S5 Correlations among elements, cytokines, and bacterial load observed in BAL from DBA/2N mice exposed to PBS or environmental C39 *S. maltophilia* strain. Spearman rank correlation coefficients were calculated on data collected on days 1, 3, and 7 p.e. Significant correlations are shown in bold. * p<0.05, ** p<0.01, *** p<0.001.
(DOCX)

Table S6 Correlations among elements observed in lung tissue and BAL from DBA/2N mice exposed to PBS or CF Sm111 *S. maltophilia* strain. Spearman rank correlation coefficients were calculated on data collected on days 1, 3, and 7 p.e. Significant correlations are shown in bold. * p<0.05, ** p<0.01, *** p<0.001.
(DOCX)

Table S7 Correlations among elements observed in lung tissue and BAL from DBA/2N mice exposed to PBS or environmental C39 *S. maltophilia* strain. Spearman rank correlation coefficients were calculated on data collected on days 1, 3, and 7 p.e. Significant correlations are shown in bold. * p<0.05, ** p<0.01, *** p<0.001.
(DOCX)
