# Supplementary material for: Stenotrophomonas maltophilia Virulence and Specific Variations in Trace Elements during Acute Lung Infection: Implications in Cystic Fibrosis
Source: PLoS One. 2014 Feb 28;9(2):e88769. doi: 10.1371/journal.pone.0088769 (PMC3938418; doi:10.1371/journal.pone.0088769)
Supplement: Data S1 — Methods and Results. (DOCX) [file pone.0088769.s001.docx]

**METHODS**

**ICP-MS operating conditions.**

ICP-MS analysis was performed using the following operating conditions: forward power = 1260 W; plasma gas flow rate = 15 l/min; carrier gas flow rate = 1.13 l/min; spray chamber temperature = 2°C; sampler and skimmer cone: Ni, 1 and 0.4 mm inside diameter, respectively; sample uptake rate = 0.4 ml/min; sample depth = 7 mm; measured m/z: ^24^Mg, ^31^P, ^34^S, ^39^K, ^44^Ca, ^51^V, ^53^Cr, ^55^Mn, ^57^Fe, ^59^Co, ^60^Ni, ^65^Cu, ^67^Zn, ^77^Se, and ^85^Rb,; sampling period per mass = 0.3 sec (three replicates). The operating conditions were optimized for the best sensitivity of 10 mg/l of ^7^Li, ^89^Y and ^205^Tl and for the reduction of ^140^Ce^16^O^+^/^140^Ce^+^ and ^140^Ce^2+^/^140^Ce^+^ ratios below 1 and 3%, respectively. The sample introduction system was washed among different analyses with 3% HNO_3_. The external standard method was used for quantification. HNO_3_ concentration of external standards was matched with acid concentration of diluted samples. Internal standard correction was performed by online addition in a T-piece of an internal standard solution of Y (50 mg/l, Agilent Technologies). Data analysis was performed with ICP-MS Chemstation software (Agilent Technologies).

**Analytical figures of merit.**

Limit of detection (LOD) and limit of quantification (LOQ) were expressed as signals (yD and yQ) on the basis of the mean value ($\mathop{y_{b}}\limits^{-}$) and standard deviation (s_b_) of the blank responses (n=10):

y_D_ = $\mathop{y_{b}}\limits^{-}$ + 2ts_b_ y_Q_ = $\mathop{y_{b}}\limits^{-}$ + 10s_b_

where t is the constant from a one-sided Student’s t-test at 95% confidence level and for n – 1 degrees of freedom. The corresponding concentration values were obtained by using an appropriate calibration curve satisfying the relationship: 0.5x1<LOD<x1, where x1 is the concentration of the first calibration level [1].

Linearity was studied according to IUPAC guidelines. Homoscedasticity and linearity of the calibration functions were verified by applying Hartley test and Mandel’s fitting test, respectively. A two-sided t-test was used to confirm the significance of the intercept. The precision was calculated in terms of repeatability as relative standard deviation (RSD%).

Trueness was evaluated verifying the possible matrix effect as proportional and/or constant systematic errors by calculating the recovery function over the entire working range [2]. The calculated external calibration function:

y = b_0ex_+ b_1ex_x_ex_

was used to quantify digested lung samples spiked with known amounts of both analytes (S_i_) and internal standard.

The founded concentrations (x_f_), calculated from the measured signals (y_f_)

x_f_ = y_f_ – (b_0ex_/b_1ex_)

were used to calculate the recovery function by plotting x_f_ versus S_i_

x_f_ = b_0f_ + b_1f_S_i_

If both the constant and proportional systematic errors are negligible, the calculated intercept (b_0f_) and the slope (b_1f_) of the recovery function will not differ significantly from 0 and 1, respectively, at a defined confidence level.

**RESULTS**

**Development of ICP-MS element profiling of lung tissue: assessment of analytical figures of merit.**

Since ICP-MS analysis has never been considered, at the best of our knowledge, for assessing the multi-element profiling of mouse respiratory specimens, in a preliminary phase of this study we investigated some pre-analytical factors and assessed the analytical performances of the ICP-MS analysis. In order to verify the sensitivity and accuracy of the proposed method, we evaluated LOD, LOQ, linearity of calibration functions, repeatability, and trueness. In particular, we checked for the suitability of the external standard method for element quantification in lung tissue. In fact, lungs were homogenized in 0.9% NaCl resulting in a significant increase of Na^+^ and Cl^-^ concentrations in the real samples compared to the external standard solutions and it is well known that Na^+^ and Cl^-^ are sources of spectral and non-spectral interferences affecting the analytical accuracy for several elements. Our data show that the homoscedasticity over the investigated calibration ranges and the linearity of the calculated calibration functions were verified for all analytes by Hartley and Mandel test, respectively (Table S1). The calculated LOD and LOQ also suggest that the method may provide suitable sensitivities for Mg, P, S, K, Ca, Mn, Fe, Co, Cu, Zn, Se, and Rb (Table S1). In contrast, the lung concentrations of V and Cr were below their LOQ values (0.1 and 16 µg/l, corresponding to 0.08 and 9 µg/g of dry tissue, respectively). V and Cr are severely affected by chloride based spectral interferences that cannot be resolved by low resolution ICP-MS. The high concentration of NaCl in the homogenized lung samples strongly decreases the method sensitivity for these elements that were, therefore, excluded from further analysis. Furthermore, acceptable repeatability and trueness were calculated for all target analytes (Table S1). In particular, the slopes of the calculated recovery functions did not significantly differ from 1 suggesting negligible proportional systematic error. However, particular attention should be made to remove blood contamination from lung tissue. In fact, we observed that animal perfusion before collecting samples for ICP-MS analysis is mandatory, since in not perfused mice blood contamination significantly increased - ranging from 25 to 88% - pulmonary concentrations of Mg, S, K, Ca, Mn, Fe, Cu, Zn, and Rb, compared with perfused ones (Figure S1).

**REFERENCES**

1. The Fitness for Purpose of Analytical Methods. In A Laboratory Guide to Method Validation and Related Topics, Eurachem Guide (1st ed.). LGC Ltd.: Teddington, 1998.

2. Funk W, Dammann V, Donnovert G (2007) Quality Assurance in Analytical Chemistry: Application in Environmental, Food, and Material Analysis, Biotechnology and Medical Engineering. Wiley-VCH: Weinheim.
