# Supplementary material for: Stenotrophomonas maltophilia Virulence and Specific Variations in Trace Elements during Acute Lung Infection: Implications in Cystic Fibrosis
Source: PLoS One. 2014 Feb 28;9(2):e88769. doi: 10.1371/journal.pone.0088769 (PMC3938418; doi:10.1371/journal.pone.0088769)
Supplement: Table S1 — ICP-MS analysis of mouse lung tissue: analytical figures of merit. The m/z measured for quantification, the internal standard (IS), the values of limit of detection (LOD) and limit of quantization (LOQ), the external calibration functions, precision and trueness are shown. Spike levels were: 20, 50, and 200 µg/l for Mg; 300, 500, and 1000 µg/l for P, S, and K; 20, 50, and 100 µg/l for Ca and Fe; 5, 20, and 100 µg/l for Cu and Rb; and 1, 5, and 10 µg/l for Mn, Co, and Se. a µg/l (µg/g); b µg/l; c CI = confidence interval, 95% confidence level; d 95% confidence level; e Coefficient of variation (%); f Standard deviation; g Relative standard deviation (%). (DOCX) [file pone.0088769.s004.docx]

| **Element (IS)** | **Calibration function** | | | | | | | | | **Repeatability**  **(CV^e^, n=5)** | | | **Trueness**  **(R%±SD^f^, n=5)** | | | **Recovery function**  **(mean±CI^d^, n=5)** | |
| --- | --- | --- | --- | --- | --- | --- | --- | --- | --- | --- | --- | --- | --- | --- | --- | --- | --- |
|  | **m/z** | **LOD^a^** | **LOQ^a^** | **Linear Range^b^** | **b0±CI^c^** | **b1±CI^c^** | **r^2^** | **Hartley test, p^d^** | **Mandel test, p^d^** | **S1, mean**  **(SD^f^, RSD^g^)^c^** | **S2, mean**  **(SD^f^, RSD^g^)^c^** | **S3, mean**  **(SD^f^, RSD^g^)^c^** | **S1** | **S2** | **S3** | **b0** | **b1** |
| Mg (^89^Y) | 24 | 5 (3) | 7 (4) | 10-500 | -2±2 | 8.50±0.02 | 0.999 | 0.058 | 0.368 | 85 (5, 6) | 118 (1, 1) | 272 (8, 3) | 87±30 | 100±4 | 102±4 | 66.3±0.4 | 1.02±0.05 |
| P (^89^Y) | 31 | 5 (3) | 15 (8) | 100-2000 | 2±15 | 2.63±0.01 | 0.997 | 0.163 | 0.864 | 578 (17, 3) | 782 (62, 8) | 1281 (47, 4) | 96+-8 | 99±13 | 99±5 | 275±5 | 1.01±0.01 |
| S (^89^Y) | 34 | 114 (64) | 402 (225) | 400-1500 | 417±8 | 0.272±0.009 | 0.978 | 0.247 | 0.561 | 575 (44, 8) | 729 (16, 2) | 1335 (22,2) | 108±24 | 88±19 | 104±10 | 251±9 | 1.0±0.2 |
| K (^89^Y) | 39 | 4 (2) | 37 (21) | 100-1500 | 5196±300 | 58.9±0.4 | 0.996 | 0.998 | 0.54 | 427 (10, 2) | 764 (50, 7) | 1270 (78,6) | 98+-9 | 98±9 | 100±8 | 344±5 | 1.01±0.01 |
| Ca (^89^Y) | 43 | 6 (3) | 14 (8) | 20-200 | 0.44±0.01 | 0.0281±0.0002 | 0.997 | 0.972 | 0.326 | 37 (1, 4) | 68 (3, 5) | 118 (7, 6) | 105±2 | 101±7 | 100±12 | 18.5±0.9 | 0.99±0.02 |
| Mn (^89^Y) | 55 | 1 (0.6) | 2 (1) | 2.0-20 | 2.6±0.4 | 13.14±0.01 | 0.998 | 0.050 | 0.415 | 2.31 (0.05, 2.0) | 6.9 (0.1, 2.1) | 12.3 (0.1, 1.7) | 95±5 | 111±3 | 103±5 | 1.455±0.002 | 1.01±0.03 |
| Fe (^89^Y) | 57 | 0.3 (0.2) | 0.7 (0.4) | 5-200 | 3.76±0.06 | 0.2732±0.0007 | 0.999 | 0.152 | 0.689 | 47 (3, 7) | 78 (2, 3) | 125 (8, 6) | 100±10 | 100±5 | 97±9 | 29.0±0.4 | 0.97±0.05 |
| Co (^89^Y) | 59 | 0.06 (0.03) | 0.1 (0.06) | 0.1-10 | -0.01±0.05 | 10.44±0.05 | 0.998 | 0.176 | 0.212 | 1.09 (0.02, 2.1) | 5.4 (0.2, 3.1) | 10.6 (0.1, 1.2) | 94±3 | 106±3 | 100±5 | 0.21±0.03 | 1.01±0.03 |
| Cu (^89^Y) | 65 | 0.7 (0.4) | 4 (2) | 5-100 | -0.4±0.3 | 2.377±0.006 | 0.999 | 0.169 | 0.702 | 16.7 (0.4, 2.2) | 30 (2, 5) | 104 (2, 2) | 108±9 | 94±8 | 93±2 | 11.9±0.1 | 0.99±0.04 |
| Zn (^89^Y) | 66 | 8 (4) | 12 (7) | 10-200 | 1.4±0.2 | 1.297±0.002 | 0.999 | 0.876 | 0.300 | 28 (2, 8) | 58 (0.1, 0.2) | 104 (5, 5) | 99±12 | 99±12 | 98±1 | 96±5 | 0.99±0.01 |
| Se (^89^Y) | 77 | 1 (0.6) | 2 (1) | 1.0-20 | 0.031±0.002 | 0.0680±0.0002 | 0.999 | 0.321 | 0.611 | 3.4 (0.3, 8.1) | 8.1 (0.2, 2.5) | 14 (1, 5) | 97±15 | 110±6 | 104±7 | 2.58±0.05 | 1.050±0.05 |
| Rb (^89^Y) | 85 | 0.05 (0.03) | 0.1 (0.06) | 5-100 | 13±3 | 8.18±0.06 | 0.996 | 0.359 | 0.999 | 23.1 (0.6, 2.7) | 40 (2, 6) | 118 (2, 2) | 118±20 | 118±9 | 101±2 | 18.2±0.2 | 1.000±0.004 |
