# Supplementary material for: Stenotrophomonas maltophilia Virulence and Specific Variations in Trace Elements during Acute Lung Infection: Implications in Cystic Fibrosis
Source: PLoS One. 2014 Feb 28;9(2):e88769. doi: 10.1371/journal.pone.0088769 (PMC3938418; doi:10.1371/journal.pone.0088769)
Supplement: Table S2 — Correlations among elements, cytokines, and bacterial load observed in lung tissue of DBA/2N mice exposed to PBS or CF Sm111 S. maltophilia strain. Spearman rank correlation coefficients were calculated on data collected on days 1, 3, and 7 p.e. Significant correlations are shown in bold. * p<0.05, ** p<0.01, *** p<0.001. (DOCX) [file pone.0088769.s005.docx]

| **Variable** | **Mg** | **P** | **S** | **K** | **Ca** | **Mn** | **Fe** | **Co** | **Cu** | **Se** | **Rb** | **CFU/mg**  **(Lung)** | **IFNγ** | **TNFα** | **IL-6** | **MIP-2** |
| --- | --- | --- | --- | --- | --- | --- | --- | --- | --- | --- | --- | --- | --- | --- | --- | --- |
| **Mg** | **1** |  |  |  |  |  |  |  |  |  |  |  |  |  |  |  |
| **P** | **0,574***** | **1** |  |  |  |  |  |  |  |  |  |  |  |  |  |  |
| **S** | **0,780***** | **0,807***** | **1** |  |  |  |  |  |  |  |  |  |  |  |  |  |
| **K** | **0,830***** | **0,508***** | **0,723***** | **1** |  |  |  |  |  |  |  |  |  |  |  |  |
| **Ca** | 0,031 | 0,067 | 0,051 | -0,059 | **1** |  |  |  |  |  |  |  |  |  |  |  |
| **Mn** | 0,124 | -0,034 | 0,080 | 0,046 | 0,242 | **1** |  |  |  |  |  |  |  |  |  |  |
| **Fe** | 0,022 | -0,068 | 0,137 | 0,048 | 0,157 | **0,448**** | **1** |  |  |  |  |  |  |  |  |  |
| **Co** | **-0,333*** | **-0,367*** | **-0,307*** | -0,210 | 0,194 | **0,644***** | **0,375**** | **1** |  |  |  |  |  |  |  |  |
| **Cu** | 0,181 | -0,035 | 0,178 | **0,336*** | 0,116 | **0,446**** | **0,314*** | **0,597***** | **1** |  |  |  |  |  |  |  |
| **Se** | **0,538***** | **0,567***** | **0,753***** | **0,467**** | 0,195 | 0,160 | 0,251 | -0,155 | **0,319*** | **1** |  |  |  |  |  |  |
| **Rb** | **0,824***** | **0,447**** | **0,688***** | **0,916***** | -0,011 | 0,100 | 0,027 | -0,137 | **0,432**** | **0,450**** | **1** |  |  |  |  |  |
| **CFU/mg** | **0,570***** | **0,694***** | **0,653***** | **0,496***** | -0,013 | **-0,393**** | -0,109 | **-0,649***** | **-0,359*** | 0,267 | **0,455**** | **1** |  |  |  |  |
| **IFNγ** | 0,163 | 0,124 | 0,164 | 0,227 | -0,140 | -0,397 | 0,159 | **-0,704**** | -0,437 | -0,095 | 0,128 | **0,824***** | **1** |  |  |  |
| **TNFα** | 0,122 | 0,137 | 0,162 | 0,179 | -0,120 | -0,438 | 0,143 | **-0,705**** | **-0,470*** | -0,085 | 0,089 | **0,822***** | **0,991***** | **1** |  |  |
| **IL-6** | 0,278 | 0,335 | 0,287 | 0,259 | -0,032 | **-0,573**** | -0,331 | **-0,889***** | **-0,598**** | 0,093 | 0,200 | **0,880***** | **0,807***** | **0,809***** | **1** |  |
| **MIP-2** | **0,468*** | **0,462*** | **0,503*** | 0,425 | 0,035 | -0,355 | -0,051 | **-0,762***** | **-0,463*** | 0,237 | 0,390 | **0,966***** | **0,863***** | **0,865***** | **0,903***** | **1** |
