# Supplementary material for: Stenotrophomonas maltophilia Virulence and Specific Variations in Trace Elements during Acute Lung Infection: Implications in Cystic Fibrosis
Source: PLoS One. 2014 Feb 28;9(2):e88769. doi: 10.1371/journal.pone.0088769 (PMC3938418; doi:10.1371/journal.pone.0088769)
Supplement: Table S3 — Correlations among elements, cytokines, and bacterial load observed in lung tissue of DBA/2N mice exposed to PBS or environmental C39 S. maltophilia strain. Spearman rank correlation coefficients were calculated on data collected on days 1, 3, and 7 p.e. Significant correlations are shown in bold. * p<0.05, ** p<0.01, *** p<0.001. (DOCX) [file pone.0088769.s006.docx]

| **Variable** | **Mg** | **P** | **S** | **K** | **Ca** | **Mn** | **Fe** | **Co** | **Cu** | **Se** | **Rb** | **CFU/mg** | **IFNγ** | **TNFα** | **IL-6** | **MIP-2** |
| --- | --- | --- | --- | --- | --- | --- | --- | --- | --- | --- | --- | --- | --- | --- | --- | --- |
| **Mg** | **1** |  |  |  |  |  |  |  |  |  |  |  |  |  |  |  |
| **P** | 0,405 | **1** |  |  |  |  |  |  |  |  |  |  |  |  |  |  |
| **S** | **0,884***** | **0,713**** | **1** |  |  |  |  |  |  |  |  |  |  |  |  |  |
| **K** | **0,896***** | **0,579**** | **0,899***** | **1** |  |  |  |  |  |  |  |  |  |  |  |  |
| **Ca** | -0,275 | -0,314 | -0,427 | -0,284 | **1** |  |  |  |  |  |  |  |  |  |  |  |
| **Mn** | 0,135 | 0,191 | 0,188 | 0,114 | 0,259 | **1** |  |  |  |  |  |  |  |  |  |  |
| **Fe** | 0,147 | 0,074 | 0,203 | 0,244 | 0,241 | **0,657**** | **1** |  |  |  |  |  |  |  |  |  |
| **Co** | **-0,492*** | -0,347 | **-0,525*** | **-0,454*** | **0,602**** | **0,657**** | **0,540*** | **1** |  |  |  |  |  |  |  |  |
| **Cu** | 0,089 | -0,214 | -0,032 | -0,033 | **0,481*** | **0,517*** | **0,528*** | **0,517*** | **1** |  |  |  |  |  |  |  |
| **Se** | **0,747***** | 0,262 | **0,653**** | **0,672**** | 0,011 | 0,143 | 0,397 | -0,229 | **0,519*** | **1** |  |  |  |  |  |  |
| **Rb** | **0,947***** | 0,290 | **0,783***** | **0,871***** | -0,179 | 0,150 | 0,275 | -0,395 | 0,137 | **0,746***** | **1** |  |  |  |  |  |
| **CFU/mg** | **0,643**** | **0,575**** | **0,739***** | **0,643**** | **-0,596**** | -0,133 | -0,181 | **-0,757***** | **-0,542*** | 0,199 | **0,554*** | **1** |  |  |  |  |
| **IFNγ** | 0,417 | 0,370 | **0,496*** | 0,377 | -0,350 | -0,132 | -0,140 | **-0,601**** | -0,413 | 0,172 | 0,356 | **0,697**** | **1** |  |  |  |
| **TNFα** | 0,419 | 0,383 | **0,497*** | 0,410 | **-0,626**** | -0,237 | -0,231 | **-0,680**** | **-0,527*** | 0,016 | 0,400 | **0,841***** | **0,577**** | **1** |  |  |
| **IL-6** | 0,159 | 0,283 | 0,232 | 0,182 | **-0,478*** | **-0,576**** | **-0,495*** | **-0,771***** | **-0,582**** | -0,063 | 0,153 | **0,620**** | **0,609**** | **0,776***** | **1** |  |
| **MIP-2** | **0,573**** | **0,601**** | **0,678**** | **0,590**** | **-0,606**** | -0,105 | -0,235 | **-0,696**** | **-0,626**** | 0,067 | **0,500*** | **0,967***** | **0,646**** | **0,857***** | **0,651**** | **1** |
