# Supplementary material for: Stenotrophomonas maltophilia Virulence and Specific Variations in Trace Elements during Acute Lung Infection: Implications in Cystic Fibrosis
Source: PLoS One. 2014 Feb 28;9(2):e88769. doi: 10.1371/journal.pone.0088769 (PMC3938418; doi:10.1371/journal.pone.0088769)
Supplement: Table S4 — Correlations among elements, cytokines, and bacterial load observed in BAL from DBA/2N mice exposed to PBS or CF Sm111 S. maltophilia strain. Spearman rank correlation coefficients were calculated on data collected on days 1, 3, and 7 p.e. Significant correlations are shown in bold. * p<0.05, ** p<0.01, *** p<0.001. (DOCX) [file pone.0088769.s007.docx]

| **Variable** | **Mg** | **Ca** | **Mn** | **Fe** | **Co** | **Cu** | **Se** | **Rb** | **CFU/mg** | **IFNγ** | **TNFα** | **IL-6** | **MIP-2** |
| --- | --- | --- | --- | --- | --- | --- | --- | --- | --- | --- | --- | --- | --- |
| **Mg** | **1** |  |  |  |  |  |  |  |  |  |  |  |  |
| **Ca** | 0,274 | **1** |  |  |  |  |  |  |  |  |  |  |  |
| **Mn** | 0,038 | **0,480**** | **1** |  |  |  |  |  |  |  |  |  |  |
| **Fe** | **0,427*** | -0,100 | -0,106 | **1** |  |  |  |  |  |  |  |  |  |
| **Co** | **-0,346*** | -0,175 | 0,154 | -0,158 | **1** |  |  |  |  |  |  |  |  |
| **Cu** | **0,583***** | 0,281 | 0,175 | 0,255 | -0,102 | **1** |  |  |  |  |  |  |  |
| **Se** | 0,255 | -0,143 | -0,095 | **0,699***** | -0,248 | 0,147 | **1** |  |  |  |  |  |  |
| **Rb** | **0,901***** | 0,078 | -0,077 | **0,356*** | -0,205 | **0,452**** | 0,126 | **1** |  |  |  |  |  |
| **CFU/mg** | **0,494**** | -0,273 | -0,170 | **0,790***** | -0,171 | 0,237 | **0,623***** | **0,405*** | **1** |  |  |  |  |
| **IFNγ** | 0,079 | -0,133 | -0,193 | **0,547**** | -0,024 | -0,181 | 0,183 | 0,007 | **0,710***** | **1** |  |  |  |
| **TNFα** | 0,042 | -0,145 | -0,300 | **0,638**** | -0,092 | -0,321 | 0,240 | -0,060 | **0,820***** | **0,898***** | **1** |  |  |
| **IL-6** | 0,047 | -0,174 | -0,253 | **0,674**** | -0,124 | -0,164 | **0,292*** | -0,086 | **0,705***** | **0,905***** | **0,952***** | **1** |  |
| **MIP-2** | 0,075 | -0,164 | -0,287 | **0,706***** | -0,111 | -0,237 | **0,349**** | -0,046 | **0,845***** | **0,862***** | **0,984***** | **0,952***** | **1** |
