# Supplementary material for: Stenotrophomonas maltophilia Virulence and Specific Variations in Trace Elements during Acute Lung Infection: Implications in Cystic Fibrosis
Source: PLoS One. 2014 Feb 28;9(2):e88769. doi: 10.1371/journal.pone.0088769 (PMC3938418; doi:10.1371/journal.pone.0088769)
Supplement: Table S5 — Correlations among elements, cytokines, and bacterial load observed in BAL from DBA/2N mice exposed to PBS or environmental C39 S. maltophilia strain. Spearman rank correlation coefficients were calculated on data collected on days 1, 3, and 7 p.e. Significant correlations are shown in bold. * p<0.05, ** p<0.01, *** p<0.001. (DOCX) [file pone.0088769.s008.docx]

| **Variable** | **Mg** | **Ca** | **Mn** | **Fe** | **Co** | **Cu** | **Se** | **Rb** | **CFU/mg** | **IFNγ** | **TNFα** | **IL-6** | **MIP-2** |
| --- | --- | --- | --- | --- | --- | --- | --- | --- | --- | --- | --- | --- | --- |
| **Mg** | **1** |  |  |  |  |  |  |  |  |  |  |  |  |
| **Ca** | **0,522**** | **1** |  |  |  |  |  |  |  |  |  |  |  |
| **Mn** | 0,069 | **0,573***** | **1** |  |  |  |  |  |  |  |  |  |  |
| **Fe** | **0,581***** | 0,098 | -0,152 | **1** |  |  |  |  |  |  |  |  |  |
| **Co** | **-0,358*** | 0,221 | **0,539**** | **-0,530**** | **1** |  |  |  |  |  |  |  |  |
| **Cu** | **0,746***** | **0,448**** | 0,005 | **0,465**** | -0,144 | **1** |  |  |  |  |  |  |  |
| **Se** | 0,144 | **-0,354*** | **-0,506**** | **0,602***** | **-0,607***** | -0,006 | **1** |  |  |  |  |  |  |
| **Rb** | **0,954***** | **0,387*** | 0,021 | **0,562**** | **-0,371*** | **0,693***** | 0,161 | **1** |  |  |  |  |  |
| **CFU/mg** | **0,590***** | 0,131 | -0,166 | **0,672***** | **-0,481**** | **0,651***** | **0,438**** | **0,549**** | **1** |  |  |  |  |
| **IFNγ** | 0,379 | -0,012 | 0,187 | **0,559*** | -0,416 | 0,364 | **0,531*** | 0,344 | **0,649**** | **1** |  |  |  |
| **TNFα** | 0,449 | 0,200 | -0,028 | **0,561*** | **-0,479*** | 0,382 | **0,519*** | 0,281 | **0,824***** | **0,546*** | **1** |  |  |
| **IL-6** | 0,119 | 0,118 | 0,028 | 0,353 | -0,283 | 0,289 | 0,396 | 0,081 | **0,590**** | **0,598**** | **0,732**** | **1** |  |
| **MIP-2** | **0,588**** | -0,157 | -0,292 | **0,718**** | **-0,722**** | **0,720**** | **0,783***** | **0,506*** | **0,975***** | **0,641**** | **0,826***** | **0,588**** | **1** |
