# Supplementary material for: Stenotrophomonas maltophilia Virulence and Specific Variations in Trace Elements during Acute Lung Infection: Implications in Cystic Fibrosis
Source: PLoS One. 2014 Feb 28;9(2):e88769. doi: 10.1371/journal.pone.0088769 (PMC3938418; doi:10.1371/journal.pone.0088769)
Supplement: Table S6 — Correlations among elements observed in lung tissue and BAL from DBA/2N mice exposed to PBS or CF Sm111 S. maltophilia strain. Spearman rank correlation coefficients were calculated on data collected on days 1, 3, and 7 p.e. Significant correlations are shown in bold. * p<0.05, ** p<0.01, *** p<0.001. (DOCX) [file pone.0088769.s009.docx]

| **Variable** | **Mg_BAL** | **Ca_BAL** | **Mn_BAL** | **Fe_BAL** | **Co_BAL** | **Cu_BAL** | **Se_BAL** | **Rb_BAL** |
| --- | --- | --- | --- | --- | --- | --- | --- | --- |
| **Mg_Lung** | **0,346*** | -0,035 | 0,070 | 0,283 | -0,315 | 0,261 | **0,552**** | 0,192 |
| **Ca_Lung** | 0,026 | 0,128 | 0,291 | -0,016 | -0,199 | 0,166 | -0,019 | -0,047 |
| **Mn_ Lung** | -0,137 | 0,087 | **0,410*** | **-0,418*** | -0,023 | -0,089 | -0,201 | -0,208 |
| **Fe_Lung** | -0,064 | -0,180 | 0,262 | **-0,357*** | 0,083 | -0,205 | -0,191 | -0,077 |
| **Co_Lung** | -0,196 | 0,184 | **0,381*** | **-0,504**** | 0,162 | -0,090 | **-0,705***** | -0,144 |
| **Cu_Lung** | -0,191 | 0,126 | 0,250 | **-0,579***** | -0,008 | -0,075 | **-0,370*** | -0,169 |
| **Se_Lung** | 0,185 | 0,213 | **0,415*** | **0,340*** | -0,205 | 0,248 | **0,494**** | -0,005 |
| **Rb_Lung** | 0,303 | -0,095 | 0,018 | 0,085 | -0,225 | 0,188 | 0,276 | 0,221 |
