# Supplementary material for: Stenotrophomonas maltophilia Virulence and Specific Variations in Trace Elements during Acute Lung Infection: Implications in Cystic Fibrosis
Source: PLoS One. 2014 Feb 28;9(2):e88769. doi: 10.1371/journal.pone.0088769 (PMC3938418; doi:10.1371/journal.pone.0088769)
Supplement: Table S7 — Correlations among elements observed in lung tissue and BAL from DBA/2N mice exposed to PBS or environmental C39 S. maltophilia strain. Spearman rank correlation coefficients were calculated on data collected on days 1, 3, and 7 p.e. Significant correlations are shown in bold. * p<0.05, ** p<0.01, *** p<0.001. (DOCX) [file pone.0088769.s010.docx]

| **Variables** | **Mg_BAL** | **Ca_BAL** | **Mn_BAL** | **Fe_BAL** | **Co_BAL** | **Cu_BAL** | **Se_BAL** | **Rb_BAL** |
| --- | --- | --- | --- | --- | --- | --- | --- | --- |
| **Mg_Lung** | 0,152 | -0,094 | -0,248 | 0,226 | **-0,393*** | 0,324 | 0,301 | 0,111 |
| **Ca_Lung** | -0,146 | 0,082 | 0,128 | -0,160 | 0,095 | 0,124 | -0,209 | -0,189 |
| **Mn_Lung** | -0,081 | -0,043 | 0,202 | -0,234 | 0,228 | 0,027 | -0,282 | -0,184 |
| **Fe_Lung** | 0,065 | 0,087 | 0,249 | -0,120 | 0,076 | -0,266 | -0,090 | 0,004 |
| **Co_Lung** | -0,201 | 0,143 | **0,401*** | **-0,528**** | **0,568***** | -0,095 | **-0,592***** | -0,279 |
| **Cu_Lung** | **-0,352*** | 0,027 | 0,267 | **-0,449**** | **0,536**** | 0,021 | **-0,543**** | **-0,408*** |
| **Se_Lung** | 0,120 | 0,098 | 0,032 | 0,025 | 0,096 | 0,300 | 0,033 | 0,044 |
| **Rb_Lung** | 0,053 | -0,016 | -0,056 | 0,149 | -0,232 | 0,247 | 0,075 | 0,018 |
