# Supplementary material for: Stenotrophomonas maltophilia Virulence and Specific Variations in Trace Elements during Acute Lung Infection: Implications in Cystic Fibrosis
Source: PLoS One. 2014 Feb 28;9(2):e88769. doi: 10.1371/journal.pone.0088769 (PMC3938418; doi:10.1371/journal.pone.0088769)
Supplement: Supplementary file 9 [file pone.0088769.s002.docx]

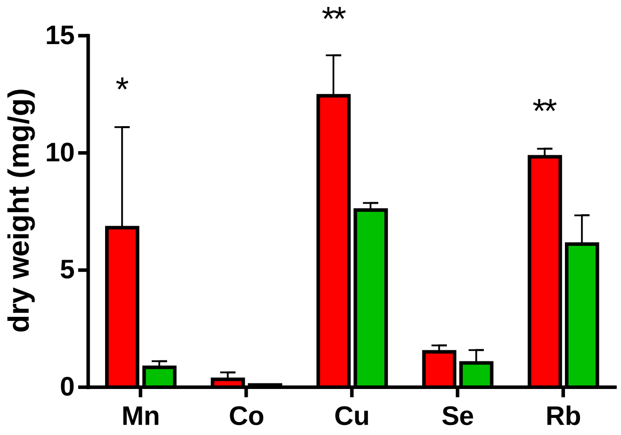

dry weight (mg/g)
15
10
5
0
*
**
**
Mn
Co
Cu
Se
Rb

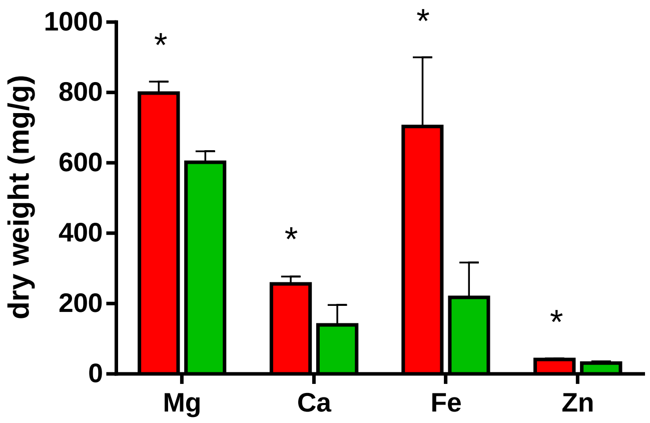

dry weight (mg/g)
1000
800
600
400
200
0
*
*
*
*
Mg
Ca
Fe
Zn

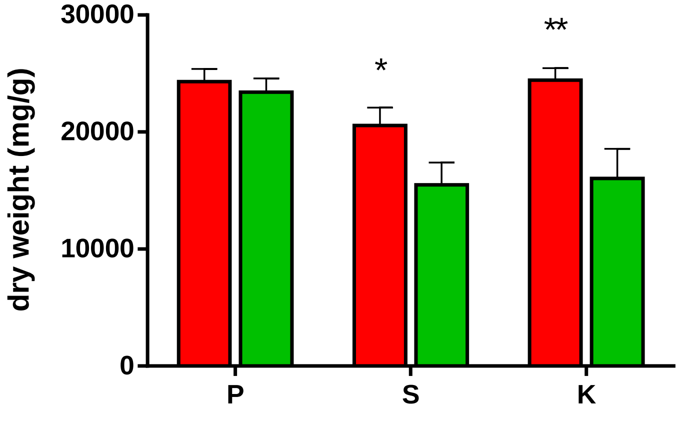

dry weight (mg/g)
30000
20000
10000
0
*
**
P
S
K
