# Supplementary material for: Stenotrophomonas maltophilia Virulence and Specific Variations in Trace Elements during Acute Lung Infection: Implications in Cystic Fibrosis
Source: PLoS One. 2014 Feb 28;9(2):e88769. doi: 10.1371/journal.pone.0088769 (PMC3938418; doi:10.1371/journal.pone.0088769)
Supplement: Supplementary file 10 [file pone.0088769.s003.docx]

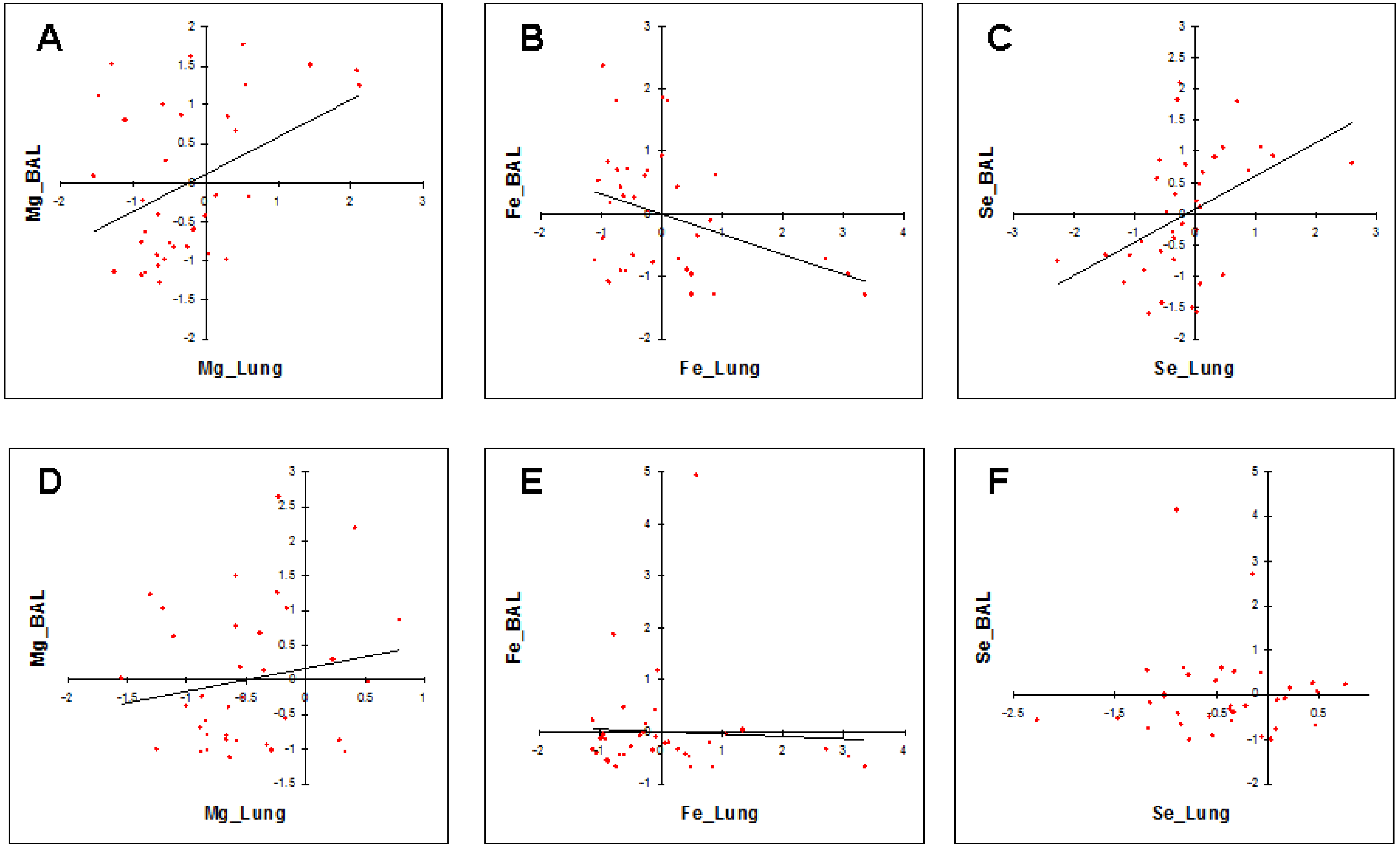
A
Mg_BAL
Mg_Lung
B
Fe_BAL
Fe_Lung
C
Se_BAL
Se_Lung
D
Mg_BAL
Mg_Lung
E
Fe_BAL
Fe_Lung
F
Se_BAL
Se_Lung
